# Protective Mechanisms of Nootropic Herb Shankhpushpi (*Convolvulus pluricaulis*) against Dementia: Network Pharmacology and Computational Approach

**DOI:** 10.1155/2022/1015310

**Published:** 2022-10-03

**Authors:** Md. Abdul Hannan, Armin Sultana, Md. Hasanur Rahman, Abdullah Al Mamun Sohag, Raju Dash, Md Jamal Uddin, Muhammad Jahangir Hossen, Il Soo Moon

**Affiliations:** ^1^Department of Biochemistry and Molecular Biology, Bangladesh Agricultural University, Mymensingh-2202, Bangladesh; ^2^Department of Pharmacy, BGC Trust University Bangladesh, Chittagong 4381, Bangladesh; ^3^Department of Biotechnology and Genetic Engineering, Faculty of Life Sciences, Bangabandhu Sheikh Mujibur Rahman Science and Technology University, Gopalganj, Bangladesh; ^4^Department of Anatomy, Dongguk University College of Medicine, Gyeongju 38066, Republic of Korea; ^5^ABEx Bio-Research Center, East Azampur, Dhaka-1230, Bangladesh; ^6^Graduate School of Pharmaceutical Sciences, College of Pharmacy, Ewha Womans University, Seoul 03760, Republic of Korea; ^7^Department of Animal Science, Patuakhali Science and Technology University, Dumki, Patuakhali 8602, Bangladesh

## Abstract

*Convolvulus pluricaulis* (CP), a *Medhya Rasayana* (nootropic) herb, is a major ingredient in Ayurvedic and Traditional Chinese formulae indicated for neurological conditions, namely, dementia, anxiety, depression, insanity, and epilepsy. Experimental evidence suggests various neuroactive potentials of CP such as memory-enhancing, neuroprotective, and antiepileptic. However, precise mechanisms underlying the neuropharmacological effects of CP remain unclear. The study, therefore, aimed at deciphering the molecular basis of neuroprotective effects of CP phytochemicals against the pathology of dementia disorders such as Alzheimer's (AD) and Parkinson's (PD) disease. The study exploited bioinformatics tools and resources, such as Cytoscape, DAVID (Database for annotation, visualization, and integrated discovery), NetworkAnalyst, and KEGG (Kyoto Encyclopedia of Genes and Genomes) database to investigate the interaction between CP compounds and molecular targets. An *in silico* analysis was also employed to screen druglike compounds and validate some selective interactions. ADME (absorption, distribution, metabolism, and excretion) analysis predicted a total of five druglike phytochemicals from CP constituents, namely, scopoletin, 4-hydroxycinnamic acid, kaempferol, quercetin, and ayapanin. In network analysis, these compounds were found to interact with some molecular targets such as prostaglandin G/H synthase 1 and 2 (*PTGS1* and *PTGS2*), endothelial nitric oxide synthase (*NOS3*), insulin receptor (*INSR*), heme oxygenase 1 (*HMOX1*), acetylcholinesterase (*ACHE*), peroxisome proliferator-activated receptor-gamma (*PPARG*), and monoamine oxidase A and B (*MAOA* and *MAOB*) that are associated with neuronal growth, survival, and activity. Docking simulation further confirmed interaction patterns and binding affinity of selected CP compounds with those molecular targets. Notably, scopoletin showed the highest binding affinity with PTGS1, NOS3, PPARG, ACHE, MAOA, MAOB, and TRKB, quercetin with PTGS2, 4-hydroxycinnamic acid with INSR, and ayapanin with HMOX1. The findings indicate that scopoletin, kaempferol, quercetin, 4-hydroxycinnamic acid, and ayapanin are the main active constituents of CP which might account for its memory enhancement and neuroprotective effects and that target proteins such as PTGS1, PTGS2, NOS3, PPARG, ACHE, MAOA, MAOB, INSR, HMOX1, and TRKB could be druggable targets against dementia.

## 1. Introduction

Dementia is a leading cause of disability and dependency among the elderly. Dementia patients may have difficulty remembering, thinking critically, behaving normally, and even performing normal daily activities. Neurodegenerative diseases (NDD) such as Alzheimer's (AD) and Parkinson's (PD) disease account for 60–80% of all dementia cases. The pathobiology of NDD is still unclear, however, pathogenic events such as oxidative stress, inflammation, apoptosis, and mitochondrial dysfunction play a critical role in the onset and progression of NDD [1]. Targeting cellular pathways that are associated with these pathological phenomena constitutes a prospective therapeutic strategy in the management of NDD. Having complex pathobiology, NDD can be adequately treated through a multitarget/multidrug therapeutic protocol [2]. With diverse phytochemical profiles, medicinal herbs are the native multidrug formulation and are utilized in many traditional therapies with no/minimal side effects [2].

*Convolvulus pluricaulis* Choisy (synonym, *Convolvulus prostratus* Forssk, belongs to Convolvulaceae) is a perennial herb native to the Indian subcontinent. Commonly termed as *Shankhpushpi* in Ayurveda*, C. pluricaulis* (CP) has been indicated for various human ailments, including those affecting the central nervous system, namely, anxiety, depression, epilepsy, and dementia [3, 4]. The pharmacological attributes owing to the health benefits of CP include anti-inflammatory, antioxidant, and immunomodulatory properties [5]. CP has been endowed with several potential phytochemicals, namely, flavonoids (kaempferol and quercetin), coumarins (scopoletin and ayapanin), phenolic acid (hydroxycinnamic acid), and phytosterol (*β*-sitosterol) that are related to its pharmacological effects [6].

A growing body of preclinical evidence has emerged supporting the ethnopharmacological uses of CP for neurological problems [7]. In healthy rats, CP extract can promote memory capacity by modulating synaptic plasticity in hippocampus [8]. The nootropic effect of CP was also confirmed by other studies [9, 10]. In various experimental models, CP can protect against neuronal injury and ameliorate memory deficits [11–15]. CP treatment prevented protein and mRNA expressions of tau and amyloid precursor protein (APP) in scopolamine-induced rat brain [16]. In drosophila model of AD, CP can rescue neurons from tau-induced neurotoxicity by attenuating oxidative stress and restoring the depleted AChE activity [17]. Scopoletin, a coumarin of CP, attenuated oxidative stress-mediated loss of dopaminergic neurons and increased the efficacy of dopamine in PD model [18]. Scopoletin also ameliorated amnesia in scopolamine-induced animals [19]. In rat model of cerebral ischemic reperfusion injury, CP improved brain pathology by antioxidant mechanism [20]. Polyherbal formulation containing CP can improve streptozotocin-induced memory deficits in rats by downregulating the mRNA expression of mitochondria-targeted cytochromes [21]. CP also improved the disease outcomes of diabetes, which are often complicated by cognitive deficits [22]. In addition, CP improved anxiety, depression, and epileptic seizure [9, 23–27]. CP can also help withstand stress conditions in experimental animals [28, 29].

The neuropharmacological effects highlighted above are mostly cumulative effects of CP phytochemicals. The existing literature, however, can hardly explain precise mechanisms that underlie the neuroactive functions of CP. Understanding the underlying molecular mechanisms through an experimental approach requires intensive endeavors. Alternatively, network pharmacology is a promising bioinformatics tool that can predict the active phytochemicals and the molecular targets that are associated with the pharmacological actions of plant extracts [30, 31]. The results obtained from Network Pharmacology could lead to further precise research in vivo. In this study, a network pharmacology and docking approach was used to explore the pharmacological mechanisms of CP phytochemicals against dementia disorders. The present study also provides evidence that helps understand the mechanisms underlying the reputed memory-enhancing capacity of CP and provides some valuable insights to advance future research to encourage the use of CP and its metabolites in the management of dementia disorders.

## 2. Materials and Methods

### 2.1. Retrieval of Compounds' Information

CP compounds were collected from the Traditional Chinese Medicine Systems Pharmacology (TCMSP) database [32]. We also verified compounds' information through PubMed database. The chemical information of CP compounds was obtained from PubChem and ChEMBL databases.

### 2.2. Compound Screening

Drug-likeness of CP compounds were predicted by QikProp (Schrödinger Release 2019–3: QikProp, Schrödinger, LLC, New York, NY, 2019). The screening was carried out based on #stars (0–5), which indicates the number of properties that fall outside the 95% range of similar values for known drugs. A compound with fewer stars is more druglike than compounds with large stars.

### 2.3. Target Retrieval

Target information for the individual compound was retrieved from TCMSP database [32]. The protein data, namely, standard protein name, gene ID, and organism were verified through UniProt (http://www.uniprot.org/) [33].

### 2.4. Network Construction

First, the individual list of AD, PD, and dementia-related genes was retrieved from DisGeNET database v6.0 [34]. Targets associated with AD, PD, and dementia are those that were common to compounds' targets. The overlapping targets amongst the lists of targets related to CP compounds, AD, PD, and dementia were obtained by the Venny 2.1.0 online software (https://bioinfogp.cnb.csic.es/tools/venny/index.html). An interaction network among compounds, targets, and diseases was established by Cytoscape v3.8.2 [35]. The nodes and edges in the network represent molecules (compounds and targets), and intermolecular interactions (compounds and targets interactions), respectively.

### 2.5. Gene Ontology (GO) Analysis

Functional enrichment analysis of Gene ontology (GO) for biological process, molecular function, and cellular components was carried out using DAVID 6.8 Gene Functional Classification Tool [36] (https://david.ncifcrf.gov/home.jsp). GO terms with a *P*value of <0.01 were considered significant. Target proteins were categorized by the Panther classification system [37] (http://pantherdb.org/).

### 2.6. Network Pathway Analysis

A protein-protein interaction network was constructed by NetworkAnalyst [38] (https://www.networkanalyst.ca/). An interactive network connecting molecular targets and associated cellular pathways was also constructed by NetworkAnalyst. Signaling and disease pathways highlighting the targets of CP compounds were retrieved through the KEGG pathway mapper [39] (https://www.genome.jp/kegg/tool/map_pathway2.html).

### 2.7. Molecular Docking and Binding Energy Analysis

#### 2.7.1. Preparation of Ligand

For virtual screening, five compounds of 2D structure with SDF format were retrieved from the PubChem database (https://pubchem.ncbi.nlm.nih.gov/) and then using ligand preparation by applying ligand preparation in Schrodinger 2017–1 with an OPLS-3 force field [40]. Before minimization, the ionization state of each compound was fixed at pH 7.0 ± 2.0 by Epik 2.2 tool [41, 42]. During the process, a maximum of 32 possible stereoisomers for every compound was generated, from where we preferred only the conformer compasses with the least energy for subsequent analysis.

#### 2.7.2. Prediction of Molecular Docking between Active Compound and Target Protein

The target proteins were downloaded from Protein Data Bank (https://www.rcsb.org/, Supplementary Table S1), were prepared and refined with the assistance of a protein preparation wizard (Schrödinger 2017–1), where the bond orders, charges, and proper hydrogen were assigned to the crystal structure. Besides, the protein structure was optimized at neutral pH by removing all nonessential water molecules. A grid box was generated automatically for Glide XP docking. Ligands and receptors were then docked by ligand docking in maestro.

#### 2.7.3. Prime MM-GBSA Analysis

Binding free energy calculation is commonly applied to analysis for determining the sum of energy produced during the binding or docking of ligand compounds with a protein [43]. The protein-ligand pose viewer file was used. In MM-GBSA (molecular mechanics with generalized born and surface area solvation) analysis, binding free energy was calculated using OPLS_3 force field as molecular mechanics energies (EMM); for polar solvation, the SGB solvation model GSGB was used, and for nonpolar solvation (GNP), Vander Waals interaction, and nonpolar solvent accessible surface area [44]. The dielectric solvent model VSGB 2.0 was used to predict the directionality of hydrogen bond and *π*-stacking interactions [43]. A higher negative binding score denotes tremendous binding.

#### 2.7.4. The Total Binding Free Energy

(1) $$\begin{array}{c} \Delta G\text{bind}=G\text{complex}-\left(G\text{protein}+G\text{ligand}\right),\;\text{where}G=\text{EMM}+\text{GSGB}+\text{GNP}. \end{array}$$

**Figure 1:**
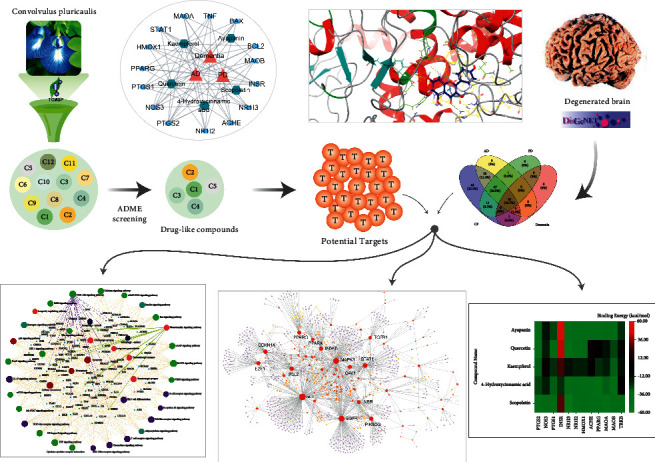
An outline of network pharmacology-based deciphering neuropharmacological mechanism of *C pluricaulis* compounds.

The flowchart of the integrated network pharmacology and *in silico* approach employed in this study is illustrated in Figure 1.

## 3. Results

### 3.1. ADME Screening

Twelve phytochemicals belonging to CP were retrieved from the TCMSP database. ADME screening offered 11 compounds having a #stars score ≤5 (Supplementary Table S2). Of these, six compounds lacking biological targets were omitted. Finally, five were chosen for further bioinformatic analysis, as displayed in Table 1. Most of the compounds are considered druglike and are more likely to be available orally as they maximally obeyed Lipinski's rule of five [45] (mol_MW < 500, QPlogPo/w < 5, donorHB°≤ 5, accptHB ≤ 10) and Jorgensen's rule of three [46] (QPlogS > −5.7, QP PCaco > 22 nm/s, # Primary Metabolites < 7), respectively. Moreover, all compounds fall within the recommended range (−3.0 to 1.2) of predicted brain/blood partition coefficient (QPlogBB) (Supplementary Table S2).

### 3.2. Target Fishing

A total of 174 possible targets of five compounds were obtained from TCMSP database (Supplementary Table S3) and validated using a literature scan in the PubMed database. Of these, a total of 117, 109, and 51 targets were found to be associated with AD, PD, and dementia, respectively, after comparing with DisGeNET database (Supplementary Table S4).

### 3.3. Network Building

**Figure 2:**
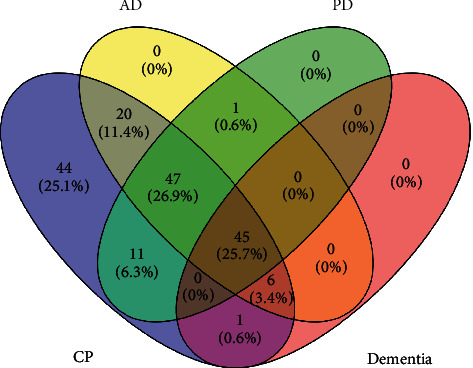
Network analysis. (a) Overlapping target genes among CP compounds, AD, PD, and dementia. (b) Compound-target-disease (C-T-D) network shows the interaction among CP compounds, targets, and dementia disorders. Hexagonal nodes represent CP compounds, whereas oval nodes represent their targets. Node size is proportional to its degree. The nodes of the first tier represent the targets with a higher degree of interaction with the compound.

Compound-target-disease (C-T-D) network established through Cytoscape could explain the multitarget effects of CP, which are used to treat brain disorders associated with cognitive deficits. C-T-D network represents the interaction of CP compounds with the targets that are linked with AD, PD, and dementia (Figure 2). Focusing on the degree of connectivity, we assume that quercetin (degree, 144) and kaempferol (degree, 58) could potentially contribute to the management of cognitive disorders. Of the targets, *PTGS1* and *PTGS2* (each with degree, 5) had the highest degree of connectivity with the compounds, followed by *NOS3, INSR, NR1I3, NR1I2, HMOX1, ACHE, PPARG, MAOA, and MAOB* (each with degree ≥3) suggesting the implication of these gene products as a prospective drug-target for CP compounds in the dementia management. The protein-protein interaction network illustrates the target proteins, some of which are direct targets of CP compounds and others are interacting proteins (Supplementary Figure S1).

### 3.4. GO Analysis

**Figure 3:**
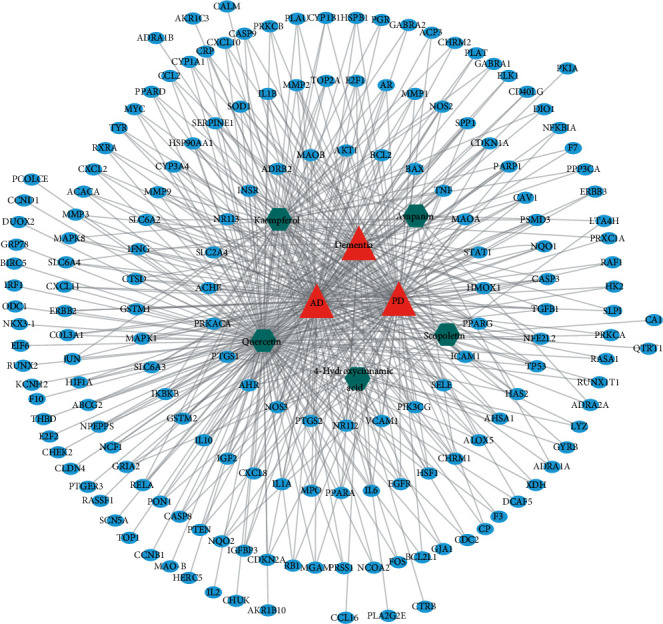
Venn diagram. Overlapping target genes among CP compounds, AD, PD, and dementia.

**Figure 4:**
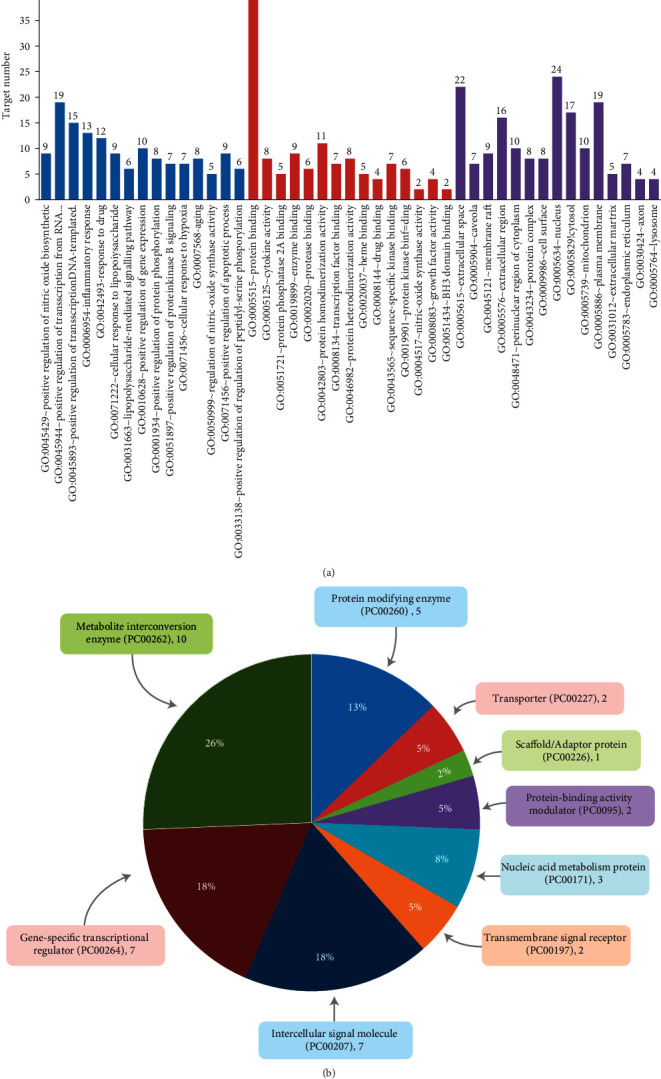
Bioinformatics analysis of overlapping target genes. (a) Gene ontology (GO) analysis: Top 15 GO terms for biological processes, molecular function, and cellular components were displayed where the *x*-axis represented GO terms for the target genes, and the *y*-axis showed target counts. The number on the tip of each bar represents the corresponding target number. Cut off: *P* < 0.001 and FDR < 0.001. (b) Panther classification categorized target proteins into nine classes. The figures next to the group in the pie chart indicate the number and percentage of protein in the given functional class.

GO analysis was carried out only with the disease-associated genes (a total of 45) that are common to AD, PD, and dementia as retrieved by employing Venny 2.1.0 online software (Figure 3). The top 15 highly enriched GO terms under biological process (BP), molecular function (MF), and cellular components (CC) (*P* < 0.05, *P*values were adjusted using the Benjamini‒Hochberg procedure) are shown in Figure 4(a). The top biological processes, including inflammatory response, response to drug, and aging have been linked to the pathophysiology of the disease, assuming that CP and its metabolites may interfere with the AD progression via modulating these biological processes. Moreover, the functional classification of target proteins suggests their diversity in biological functions (Figure 4(b)).

### 3.5. Analysis of Cellular Pathways and Targets Involved in the Pathobiology of Dementia Disorders

**Figure 5:**
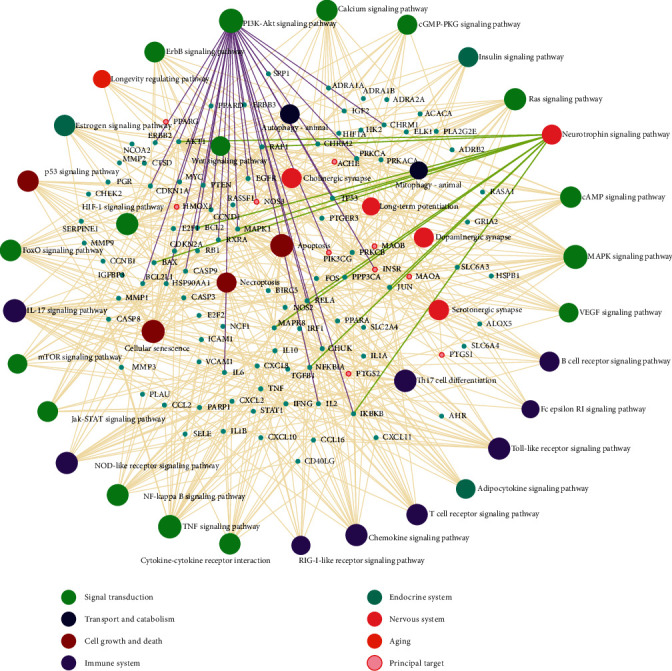
Integrated target-pathway network, a comprehensive network that visualizes the interactions of curcumin's targets with cellular pathways, which were categorized into seven modular systems (differentiated by color) using KEGG pathway annotation. Potential druggable targets were marked with small pink circles.

An interactive network illustrates top cellular pathways that involved targets of CP compounds (Figure 5). Cellular pathways were grouped into various modular systems according to KEGG pathway annotation.

**Figure 6:**
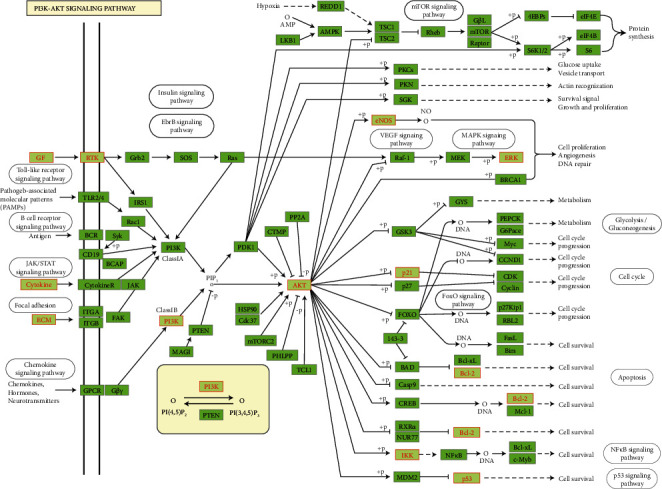
PI3K-Akt pathway is a top enriched signaling pathway. CP targets are highlighted in red.

Among the signaling pathways that were enriched (Adjusted *P*value <0.05) in the “signal transduction” module (Figure 5), the highly enriched pathway was PI3K/Akt signaling, followed by MAPK signaling, which is critically implicated in neuronal maturation and survival. PI3K/Akt pathway retrieved from KEGG pathway database illustrates a total of 12 targets that were targeted by the CP compounds (Figure 6). The upstream signaling receptor to PI3K/Akt pathway is TrkB which bound to the natural ligand, namely, brain-derived neurotrophic factor (BDNF) conveys neurotrophin signals to several downstream effectors such as Bcl-2 and Bax. Based on this information, it was further verified by docking analysis whether the CP compounds could interact with the TrkB.

Among the endocrine system-related pathways, insulin receptor signaling was the top overrepresented pathway. Insulin receptors (*INSR*) were highly connected by CP compounds, and their interaction was further verified by molecular docking. Several signaling pathways related to inflammation including TNF pathway, HIF-1 pathway, and NF-*κ*B pathway were enriched (Figure 5). Since cyclooxygenases such as COX-1 and COX-2 (*PTGS1* and *PTGS2*) catalyzing the production of inflammatory mediators were targeted by CP compounds with the highest degree of connectivity (Figure 3), their interaction was further verified by docking simulation.

In addition, nervous system-related pathways such as neurotrophin signaling pathway, cholinergic synapse, dopaminergic synapse, serotonergic synapse, and long-term potentiation were enriched (Figure 5). Any abnormality in these pathways disrupts brain function leading to the onset of NDD and related pathology. Notably, acetylcholinesterase (ACHE) has clinical significance in cholinergic deficits and therefore its binding and interaction with CP compounds were further verified with docking analysis. A number of immune system-related pathways, namely, tolllike receptor, T cell and B cell receptor, chemokine, and NODlike receptor signaling pathways were also highlighted in the network (Figure 5).

**Figure 7:**
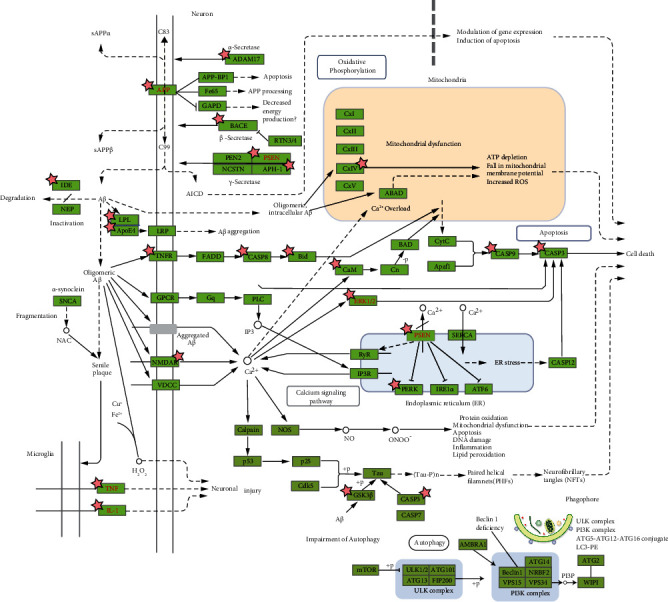
KEGG pathway of Alzheimer's disease. Targets of CP compounds are marked with asterisk (*∗*). Of these, *β*-secretase and GSK-3*β* are the potential druggable targets for AD therapy.

An AD-pathway (Figure 7) was retrieved from KEGG pathway database, illustrating a total of 13 proteins including those that are involved in amyloidogenesis (for example, APP and PSEN), cellular survival, and growth (for example, INSR, Akt, and Erk1/2) and inflammation (for example, iNOS, COX2, IKK, TNF, IL-1, and IL-6), which are potential targets of CP compounds as appeared in network pharmacology. Considering the appearance of INSR and COX2 in network pharmacology and in AD pathobiology, their interactions with the selected CP compounds were further verified by docking simulation. In addition, monoamine oxidases (MAOA and MAOB) are potential targets for both AD and PD, and thus their interactions with the selected CP compounds were also further verified.

### 3.6. *In Silico* Analysis

We employed molecular docking analysis to validate the interaction patterns and the efficiency of CP phytochemicals with some of the vital target proteins that showed a higher degree of connectivity in network pharmacology. Accordingly, we selected *PTGS2, NOS3, PTGS1, INSR, NR1I3, NR1I2, HMOX, ACHE, PPARG, MAOA,* and *MAOB* for further analysis. Additionally, we included TrkB in docking analysis since several downstream effectors of TrkB receptor signaling, including PI3K, AKT1, BAX, and BCL2, showed a higher degree of connectivity in the network (Figure 2), and TrkB is a potential receptor for neuronal growth and survival.

**Figure 8:**
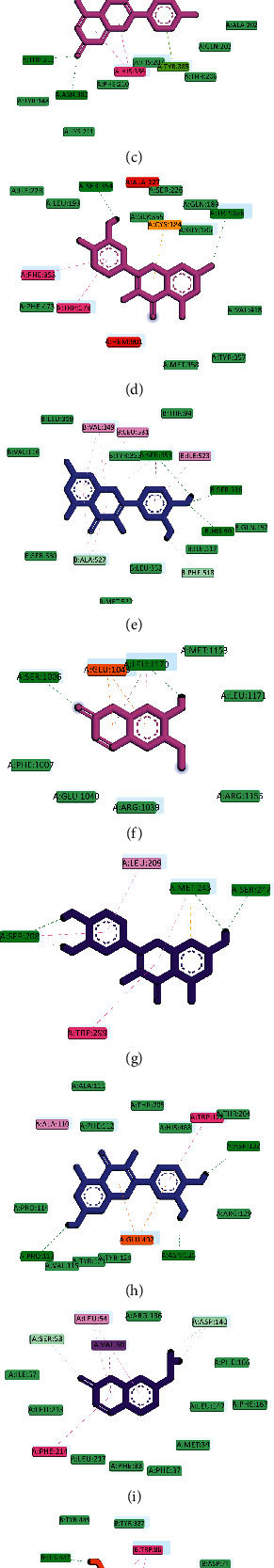
Molecular docking analysis of target proteins and compounds. Heatmap representing the binding energy revealed from MM-GBSA analysis (a). Two-dimensional molecular interaction for protein-ligand complex for TRKB-Scopoletin (b), PTGS2-Quercetin (c), NOS3-Scopoletin (d), PTGS1-Scopoletin (e), INSR-4-hydroxycinnamic acid (f), NR1I3-Scopoletin (g), NR1I2-Scopoletin (h), HMOX1-Ayapanin (i), AChE-Scopoletin (j), PPARG-4-Hydroxycinnamic acid (k), MAOA-Scopoletin (l), and MAOB-4-Hydroxycinnamic acid (m).

In any docking analysis of protein-ligand, it is ascertained that if the predicted complex obtained docking scores less than zero, indicating binding affinity of the ligand toward the receptor. However, molecular docking usually used approximated scoring functions to calculate binding energies, which are not correlated with experimental values [47, 48]. In such a case, we used MM-GBSA binding energy calculation to compute the free energy of binding the complex, which uses an implicit continuum solvent approximation [49]. A total of five compounds, namely, scopoletin, 4-hydroxycinnamic acid, kaempferol, quercetin, and ayapanin, were subjected to molecular docking to the corresponding proteins of 12 target genes (*PTGS2, NOS3, PTGS1, INSR, NR1I3, NR1I2, HMOX, ACHE, PPARG, MAOA, MAOB,* and *TRKB*), and the obtained docked complex was further subjected for MM-GBSA analysis. As shown in Figure 8, the quercetin-PTGS2 complex represented the highest binding energy of −46.27 kcal/mol, while in NOS3, the scopoletin showed maximum binding affinity and formed a stable complex with a binding energy of −34.98 kcal/mol. Interestingly, scopoletin also showed maximum binding energy to form complexes with PTGS1, NR1I3, NR1I2, ACHE, MAOA, and TRKB with binding energies of −36.28, −56.01, −39.13, −43.13, −51.18, and −34.67 kcal/mol, respectively. On the other hand, while bound to INSR, MAOB, and PPARG, 4-hydroxycinnamic acid showed maximum binding energies of −21.46, −34.044, and −41.04 kcal/mol, respectively. In HMOX1, ayapanin showed higher binding energy than other compounds. The details of molecular interactions of top hits from docking analysis are shown in Figure 8.

## 4. Discussion

Traditional knowledge and experimental evidence suggest that *C. pluricaulis*, alone or in combination, can enhance memory and protect against cognitive impairment [3, 4, 6, 50]. However, the underlying mechanisms supporting these claims remain largely unexplored. The present study, therefore, employed integrated network pharmacology and *in silico* approach to provide an in-depth insight into the neuropharmacological effects of CP phytochemicals and their protective potential against dementia. Virtual ADME screening identified a total of five active compounds from CP, such as scopoletin, 4-hydroxycinnamic acid, kaempferol, quercetin, and ayapanin showing drug-likeness and blood-brain barrier permeability. Growing evidence suggest neurorestorative and memory protective potentials of these compounds. Quercetin, a natural polyphenolic of many plants, fruits, and vegetables, is found to be effective in protecting neurons from various injuries and ameliorating cognitive deficits [51]. Quercetin can ameliorate Alzheimer's disease pathology (such as *β*-amyloidosis, tauopathy, astrogliosis and microgliosis in the hippocampus and the amygdala) and recover cognitive deficits in triple transgenic Alzheimer's disease model mice [52, 53]. Another study has shown that quercetin can ameliorate hippocampus-dependent learning and memory deficits in mice fed with high fat diet through attenuating oxidative stress by activating antioxidant signaling system [54]. The flavonoid antioxidant, kaempferol, is also equally available in fruits and vegetables showing neuroprotective effects and memory-promoting potentials in experimental models of AD, PD, and other neurological diseases [55, 56]. Kaempferol can attenuate A*β*25-35-induced apoptosis of PC-12 cells via the ER/ERK/MAPK signaling pathway [57]. Other compounds, including scopoletin and 4-hydroxycinnamic acid, were also shown to be protective against neuronal damage and effective in ameliorating memory deficits [19, 58, 59]. 4-Hydroxycinnamic acid (*P*-coumaric acid) promotes hippocampal neurogenesis, improves cognitive functions, and reduces anxiety in post-ischemic stroke rats by activating BDNF/TrkB/AKT signaling pathway [60]. Scopoletin shows neuroprotective effects by inhibiting MOA, A*β* aggregation, and lipid peroxidation [61]. Another study shows that scopoletin can attenuate intracerebral hemorrhage-induced brain injury and improve neurological performance in rats [62].

The C-T-D network illustrates that the selected CP metabolites were linked to the target proteins of dementia-associated cellular pathways. GO analysis revealed several enriched biological processes such as inflammatory response, response to drug, and aging that are implicated in the pathobiology of NDD. Network pathway analysis also shows that CP metabolites target several markers of the top enriched pathways. PI3K/Akt signaling is at the top of the enriched pathways associated with the development, survival, and activity of neurons. This pathway has multiple downstream effector targets including those associated with cell survival (Bcl-2, Bax, IKK, NF-*κ*B, and p53). Bcl-2 is a prosurvival protein whereas Bax is a proapoptotic protein. IKK, NF-*κ*B, and p53 are involved in inflammatory response [63, 64]. Other signaling pathways, particularly the MAPK pathway, in association with PI3K/Akt signaling take part in the regulation of growth and survival of cells.

Several pathways that are associated with nervous system, namely, neurotrophin signaling pathway, long-term potentiation, and cholinergic, dopaminergic, and serotonergic synapses were enriched, indicating that CP compounds may have shown neuropharmacological effects by modulating these neuronal pathways. Neurotrophin signaling pathway maintains growth, maintenance, and survival of neurons. In aging or degenerating brain, there is inadequate neurotrophic support, causing neuronal death [65]. Neurotrophin, in particular BDNF, mimetic could, therefore, have clinical importance in the management of NDD [66]. Downstream to the neurotrophin signaling is PI3K/Akt pathway, which was highly enriched in this study, and CP compounds were found to target the genes involved. As BDNF mimetic, 7,8-dihydroxyflavone, a TrkB agonist, has shown neurotrophic activities [67] and has been found to be effective in ameliorating motor and cognitive deficits [68]. Docking analysis further indicates that scopoletin exhibited the highest binding affinity to TrkB, the receptor of neurotrophin signaling pathway, and may act as a BDNF-mimetic and take part in neuronal growth and survival by modulating the classical neurotrophin/PI3K/Akt signaling.

In AD pathobiology, there is a cholinergic deficit due to dysfunction of cholinergic synapse. Although symptomatic, acetylcholinesterase (AChE) inhibitors such as donepezil, rivastigmine, and galantamine are currently in use to compensate for memory deficits due to cholinergic dysfunction [69]. Molecular docking has predicted that except for kaempferol and quercetin, the other three compounds may interrupt AChE activity. The current data suggest that these CP compounds would be a promising alternative to existing AChE inhibitors for AD patients.

Among the endocrine pathways, the dominant pathway is the insulin signaling pathway, which plays an essential role in ensuring neuronal survival and homeostasis, promoting synaptic plasticity and thereby supporting learning and memory function [70, 71]. Evidence shows that insulin signaling is impaired in degenerating brains [71]. Targeting impaired insulin signaling, therefore, constitutes a viable strategy against NDD. In docking analysis, 4-hydroxycinnamic acid showed the highest binding affinity with insulin receptor (*INSR*) although in network pharmacology quercetin and kaempferol interact with this target.

There was an enrichment of inflammation-related pathways, including TNF pathway, HIF-1 pathway, and NF-*κ*B pathway, suggesting that anti-inflammatory effects mediated by CP compounds would play a pivotal role in preventing inflammatory cascade during pathobiological progression of NDD. Cyclooxygenase enzymes, namely, COX-1 (*PTGS1*) and COX-2 (*PTGS2*) catalyze the biosynthesis of inflammatory mediators such as prostaglandins and thromboxane. In the brain, COX-2 is activated by excitatory synaptic activity in neurons and by inflammation in the glia. COX-1/COX-2 pathway has pathogenic relevance in preclinical stages of Alzheimer's disease development [72]. Pathological activation of COX-2 disrupts hippocampal synaptic function, leading to cognitive deficits [72]. Cyclooxygenase inhibitors, such as nonsteroidal anti-inflammatory drugs (NSAIDs), may have preventive effects against dementia [73]. Several COX-2 inhibitors such as celecoxib [74] and indomethacin [75] have shown promise in the management of AD. Docking results demonstrate that all CP compounds, including scopoletin and quercetin, exhibited substantial binding affinity to COX-2 and COX-1, suggesting their potential application in the development of antineuroinflammatory agents. Previous *in silico* reports on interaction of COX-2 with quercetin and kaempferol also support our data [76].

In addition to the above cellular pathways, CP compounds target some other pathways, namely, autophagy, mitophagy, apoptosis, necroptosis, and some specific molecular markers of AD and PD pathways. Endothelial nitric oxide synthase or eNOS (NOS3) is known for its outstanding role in regulating cerebral blood flow and is associated with synaptic plasticity such as long-term potentiation [77]. eNOS attenuates ischemic damage by regulating BDNF expression [78]. Nitric oxide produced by eNOS protects neurons from Tau pathology [79]. Another study reports that pharmacological activation of PI3K-eNOS signaling can ameliorate cognitive deficits in streptozotocin-induced rats [80]. Pharmacological interruption of eNOS activity results in an increase in inflammatory mediators, such as iNOS in rat ischemic brains [81]. eNOS is, thereby, protective against inflammation and other pathologic stimuli. Statins such as atorvastatin and simvastatin may contribute to the amelioration of brain tissue injury in ischemic brain by activating eNOS [82]. Together, this evidence suggests that CP compounds that target eNOS may have pharmacological significance against NDD pathobiology.

Other important targets are monoamine oxidases (MAOs) that catalyze the oxidative deamination of monoamines and contribute to the metabolism of dopamine, a neurotransmitter of dopaminergic neurons. Drugs that inhibit MAO, particularly MAOB, such as selegiline and rasagiline are currently in clinical use in patients with PD [83–85]. Docking findings demonstrate that CP compounds, particularly 4-hydroxycinnamic acid and scopoletin, showed higher binding affinity, suggesting their prospects as MAO inhibitors to be used in PD management.

Heme oxygenase-1 or HO-1 (*HMOX1*) is a stress-sensitive enzyme that catalyzes the breakdown of heme into iron, carbon monoxide, and biliverdin/bilirubin and is involved in the pathobiology of AD and other brain disorders. Astroglial induction of the *HMOX1* by *β*-amyloid and cytokines leads to mitochondrial iron sequestration and may thereby contribute to pathological iron deposition and bioenergy failure [86]. Pharmacological intervention in glial HO-1 activity may provide neuroprotection in AD by limiting iron-mediated neurotoxicity [86]. All CP compounds except kaempferol exhibit higher binding affinity to HO-1, and thereby, may be neuroprotective through regulating HO-1 activity.

Peroxisome proliferator-activated receptor-gamma or PPAR*γ* (*PPARG*), a ligand-activated nuclear transcription factor, regulates the expression of multiple genes that encode proteins involved in the regulation of lipid metabolism, improvement of insulin sensitivity, and inhibition of inflammation [87]. PPAR*γ* agonists counteract oxidative stress, neuroinflammation, and A*β* clearance [70, 88]. PPAR*γ* agonists such as fenofibrate, icariin, and naringenin are known to be neuroprotective, supporting neuronal development, synaptic plasticity, and ameliorating cognitive deficits [70, 89, 90]. In docking analysis, 4-hydroxycinnamic acid and scopoletin showed the highest binding affinity to PPAR*γ*, suggesting that these compounds can ameliorate cognitive deficits through activating PPAR*γ* signaling.

## 5. Conclusion

The *in silico* analysis predicts that CP metabolites, namely, scopoletin, 4-hydroxycinnamic acid, kaempferol, quercetin, and ayapanin are the major bioactive leads that showed interaction with various molecular targets and cellular pathways crucial to neuronal growth, survival, and activity. The signaling pathways that CP compounds primarily target include the PI3K/Akt signaling pathway, the neurotrophin signaling pathway, and the insulin signaling pathway. In addition, top targets of CP compounds including PTGS1, PTGS2, NOS3, INSR, HMOX1, ACHE, PPARG, MAOA, MAOB, and TRKB may be potential druggable targets for future drug designing to address dementia disorders. Together with the previous reports, the combined network pharmacology and in-silico observations form a scientific basis that supports the ethnomedical application of CP for memory enhancement and against aging/pathological cognitive deficits. However, further investigation of memory-enhancing and neuroprotective effects of CP and its metabolites is essential to extrapolate the findings from preclinical and *in silico* models into clinical subjects.

## Figures and Tables

**Table 1:** Druglike compounds of *C. pluricaulis* as screened by QikProp ADME prediction tool.

Compound name	Chemical nature	Structure	ADME parameters		
			^a#^stars	^b^ rule of five	^c^ rule of three
Scopoletin	Coumarin	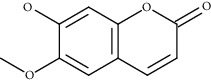	0	0	0
Hydroxycinnamic acid	Carboxylic acid	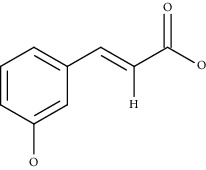	0	0	0
Kaempferol	Flavonoid	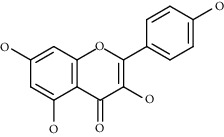	0	0	0
Quercetin	Flavonoid	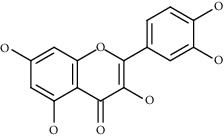	0	0	1
Ayapanin	Coumarin	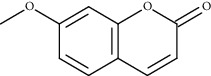	1	0	0

^a#^Stars indicates the number of property or descriptor values that fall outside the 95% range of similar values for known drugs (ranging from 0–5). A large number of stars suggests that a molecule is less druglike than molecules with few stars. The following properties and descriptors are included in the determination of #stars: MW, donorHB, accptHB, QPlogPw, QPlogPo/w, QPlogS, QPLogKhsa, QPlogBB, and #metabol. ^b^Rule of five indicates the number of violations of Lipinski's rule of five [3]. The rules are: mol_MW < 500, QPlogPo/w < 5, donor HB ≤ 5, accptHB ≤ 10. Compounds that satisfy these rules are considered druglike (maximum is 4). ^c^Rule of three indicates the number of violations of Jorgensen's rule of three. The three rules are QPlogS > −5.7, QP PCaco > 22 nm/s, # Primary Metabolites < 7. Compounds with fewer (and preferably no) violations of these rules are more likely to be orally available (maximum is 3).

## Data Availability

The data used to support the findings of the study are available in the supplementary files and additional supporting data can be obtained from the corresponding author upon request.

## Supplementary Materials

Supplementary Materials Supplementary Figure S1. Protein-protein interaction network. Node size is proportional to its degree of connectivity. Supplementary Table S1. Target genes for molecular docking. Supplementary Table S2. QikProp ADMET of CP compounds. Supplementary Table S3. Targets for CP compounds. Supplementary Table S4 data for CP compound-target-disease network. Supplementary Table S5. MM-GBSA free energy calculation result.
