# Supplementary material for: Protective Mechanisms of Nootropic Herb Shankhpushpi (Convolvulus pluricaulis) against Dementia: Network Pharmacology and Computational Approach
Source: Evid Based Complement Alternat Med. 2022 Oct 3;2022:1015310. doi: 10.1155/2022/1015310 (PMC9550454; doi:10.1155/2022/1015310)
Supplement: Supplementary Materials — Supplementary Figure S1. Protein-protein interaction network. Node size is proportional to its degree of connectivity. Supplementary Table S1. Target genes for molecular docking. Supplementary Table S2. QikProp ADMET of CP compounds. Supplementary Table S3. Targets for CP compounds. Supplementary Table S4 data for CP compound-target-disease network. Supplementary Table S5. MM-GBSA free energy calculation result. [file 1015310.f1.zip › Suppl-Table S1.pdf]

Table S1_Target genes for molecular docking

Target Name	PDB ID
PTGS2	5F19
NOS3	1M9K
PTGS1	3kk6
INSR	1GAG
NR1I3	1XV9
NR1I2	1ILH
HMOX1	1S8C
ACHE	4EY6
PIK3CG	2A4Z
PPARG	7AWD
MAOA	2Z5Y
MAOB	2BYB
