# Supplementary material for: Protective Mechanisms of Nootropic Herb Shankhpushpi (Convolvulus pluricaulis) against Dementia: Network Pharmacology and Computational Approach
Source: Evid Based Complement Alternat Med. 2022 Oct 3;2022:1015310. doi: 10.1155/2022/1015310 (PMC9550454; doi:10.1155/2022/1015310)
Supplement: Supplementary Materials — Supplementary Figure S1. Protein-protein interaction network. Node size is proportional to its degree of connectivity. Supplementary Table S1. Target genes for molecular docking. Supplementary Table S2. QikProp ADMET of CP compounds. Supplementary Table S3. Targets for CP compounds. Supplementary Table S4 data for CP compound-target-disease network. Supplementary Table S5. MM-GBSA free energy calculation result. [file 1015310.f1.zip › Suppl-Table S2.pdf]

## Table S2_QikProp ADMET of CP compounds

| molecule | #stars | #amine | #amidine | #acid | #amide | #rotor | #rtvFG | CNS |
|---|---|---|---|---|---|---|---|---|
| Scopolin | 0 | 0 | 0 | 0 | 0 | 8 | 2 | -2 |
| Convoline | 0 | 1 | 0 | 0 | 0 | 5 | 2 | 1 |
| Convolam | 0 | 1 | 0 | 0 | 0 | 4 | 1 | 1 |
| Convolvin | 0 | 1 | 0 | 0 | 0 | 4 | 1 | 1 |
| Scopoletir | 0 | 0 | 0 | 0 | 0 | 2 | 1 | 0 |
| Hydroxyci | 0 | 0 | 0 | 1 | 0 | 4 | 1 | -2 |
| Kaempfer | 0 | 0 | 0 | 0 | 0 | 4 | 0 | -2 |
| Quercetin | 0 | 0 | 0 | 0 | 0 | 5 | 0 | -2 |
| Ayapanin | 1 | 0 | 0 | 0 | 0 | 1 | 1 | 0 |
| Delphinidi | 2 | 0 | 0 | 0 | 0 | 6 | 4 | -2 |
| Taraxerol | 5 | 0 | 0 | 0 | 0 | 1 | 0 | 1 |
| β-sitoster | 6 | 0 | 0 | 0 | 0 | 7 | 0 | 0 |

a#Stars indicates the number of property or descriptor values that fall outside the 95% range
bRule of five indicates the number of violations of Lipinski's rule of five [3]. The rules are: mol_
cRule of three indicates the number of violations of Jorgensen's rule of three. The three rules

| mol_MW | dipole | SASA | FOSA | FISA | PISA | WPSA | volume | donorHB |
|---|---|---|---|---|---|---|---|---|
| 354.313 | 4.086 | 545.048 | 155.331 | 237.821 | 151.896 | 0 | 992.725 | 4 |
| 307.346 | 2.562 | 569.028 | 387.354 | 85.951 | 95.722 | 0 | 992.119 | 1 |
| 305.373 | 1.731 | 620.439 | 488.003 | 36.735 | 95.701 | 0 | 1059.573 | 0 |
| 291.346 | 1.348 | 553.508 | 403.298 | 60.676 | 89.534 | 0 | 958.939 | 1 |
| 192.171 | 7.41 | 377.41 | 89.124 | 116.981 | 171.304 | 0 | 610.457 | 1 |
| 164.16 | 3.449 | 376.676 | 25.371 | 168.922 | 182.382 | 0 | 585.13 | 2 |
| 286.24 | 4.588 | 503.264 | 0 | 237.585 | 265.68 | 0 | 839.699 | 3 |
| 302.24 | 4.899 | 514.002 | 0 | 284.18 | 229.822 | 0 | 860.567 | 4 |
| 176.171 | 5.261 | 366.516 | 91.898 | 72.055 | 202.564 | 0 | 584.993 | 0 |
| 338.701 | 1.532 | 490.29 | 160.796 | 247.136 | 56.973 | 25.385 | 720.222 | 2 |
| 426.724 | 1.906 | 684.569 | 634.721 | 36.439 | 13.409 | 0 | 1383.356 | 1 |
| 414.713 | 1.903 | 744.406 | 666.128 | 48.445 | 29.832 | 0 | 1444.693 | 1 |

of similar values for known drugs (ranging from 0 ? 5). A large number of stars suggests that a
_MW < 500, QPlogPo/w < 5, donor HB ≤5, accptHB ≤10. Compounds that satisfy these rules a
are: QPlogS > -5.7, QP PCaco > 22 nm/s, # Primary Metabolites < 7. Compounds with fewer (a

| accptHB | dip^2/V | ACxDN^.5 | glob | QPpolrz | QPlogPC1 | QPlogPoct | QPlogPw | QPlogPo/\ |
|---|---|---|---|---|---|---|---|---|
| 12.5 | 0.016816 | 0.045868 | 0.882978 | 29.889 | 11.215 | 22.842 | 20.088 | -0.949 |
| 6.7 | 0.006615 | 0.011775 | 0.845423 | 31.254 | 8.922 | 14.822 | 9.285 | 1.762 |
| 5.5 | 0.002826 | 0 | 0.810127 | 34.596 | 8.946 | 13.518 | 6.648 | 2.604 |
| 5 | 0.001895 | 0.009033 | 0.849641 | 30.509 | 8.391 | 13.482 | 7.58 | 2.368 |
| 4 | 0.089948 | 0.010599 | 0.922124 | 18.628 | 6.346 | 10.591 | 7.552 | 0.891 |
| 2.75 | 0.020334 | 0.010325 | 0.898186 | 16.436 | 6.518 | 9.807 | 7.771 | 1.419 |
| 4.5 | 0.025069 | 0.015487 | 0.855299 | 27.42 | 10.171 | 16.513 | 12.297 | 1.036 |
| 5.25 | 0.027893 | 0.020428 | 0.851249 | 27.27 | 10.666 | 18.489 | 14.379 | 0.362 |
| 3.25 | 0.047309 | 0 | 0.922939 | 18.55 | 5.762 | 7.981 | 5.45 | 1.044 |
| 1.5 | 0.003258 | 0.004327 | 0.792544 | 19.361 | 7.13 | 9.893 | 6.06 | 1.309 |
| 1.7 | 0.002627 | 0.002483 | 0.877076 | 48.694 | 11.563 | 18.2 | 4.624 | 7.066 |
| 1.7 | 0.002508 | 0.002284 | 0.830244 | 47.453 | 12.344 | 17.484 | 3.638 | 7.393 |

ı molecule is less drug-like than molecules with few stars. The following properties and descrip
re considered drug-like (maximum is 4)
ınd preferably no) violations of these rules are more likely to be orally available (maximum is 3

| QPlogS | CIQPlogS | QPlogHER | QPPCaco | QPlogBB | QPPMDCk | QPlogKp | IP(eV) | EA(eV) |
|---|---|---|---|---|---|---|---|---|
| -1.677 | -2.342 | -4.15 | 55.036 | -1.952 | 21.535 | -4.599 | 9.572 | 1.182 |
| -2.234 | -2.159 | -5.216 | 378.204 | -0.141 | 191.338 | -4.349 | 9.835 | 0.517 |
| -3.083 | -2.344 | -5.787 | 1107.751 | 0.348 | 611.317 | -3.538 | 9.372 | 0.458 |
| -2.709 | -2.494 | -5.118 | 656.771 | 0.161 | 347.437 | -4.001 | 9.371 | 0.493 |
| -1.627 | -2.203 | -3.609 | 770.14 | -0.474 | 373.031 | -2.88 | 9.343 | 0.965 |
| -1.641 | -1.812 | -2.242 | 62.749 | -1.074 | 31.561 | -3.606 | 9.13 | 0.714 |
| -3.09 | -4.073 | -5.14 | 55.32 | -1.843 | 21.655 | -4.578 | 9.124 | 0.643 |
| -2.83 | -4.043 | -5.035 | 20 | -2.352 | 7.21 | -5.467 | 9.136 | 0.657 |
| -1.38 | -1.906 | -3.745 | 2054.026 | -0.061 | 1077.06 | -2.038 | 9.264 | 0.869 |
| -3.486 | -4.774 | -5.161 | 44.907 | -2.359 | 23.808 | -5.297 | 5.874 | -0.889 |
| -7.983 | -7.261 | -3.585 | 4470.446 | 0.187 | 2496.332 | -2.048 | 9.48 | -1.096 |
| -8.121 | -7.033 | -4.421 | 3439.51 | -0.327 | 1880.353 | -1.635 | 9.521 | -0.99 |

tors are included in the determination of #stars: MW, donorHB, accptHB, QPlogPw, QPlogPo/w

)

| #metab | QPlogKhsa | HumanOra | PercentHu | SAfluorine | SAamideC | PSA | #NandO | RuleOfFive |
|---|---|---|---|---|---|---|---|---|
| 5 | -0.933 | 2 | 52.544 | 0 | 0 | 148.954 | 9 | 0 |
| 2 | -0.18 | 3 | 83.398 | 0 | 0 | 76.197 | 6 | 0 |
| 3 | 0.053 | 3 | 96.685 | 0 | 0 | 52.78 | 5 | 0 |
| 2 | 0.102 | 3 | 91.236 | 0 | 0 | 62.507 | 5 | 0 |
| 2 | -0.49 | 3 | 83.826 | 0 | 0 | 70.484 | 4 | 0 |
| 1 | -0.672 | 3 | 67.426 | 0 | 0 | 74.003 | 3 | 0 |
| 4 | -0.201 | 3 | 64.205 | 0 | 0 | 120.544 | 6 | 0 |
| 5 | -0.354 | 2 | 52.348 | 0 | 0 | 141.943 | 7 | 0 |
| 1 | -0.596 | 3 | 92.348 | 0 | 0 | 49.178 | 3 | 0 |
| 7 | -0.129 | 2 | 51.222 | 0 | 0 | 100.299 | 7 | 1 |
| 2 | 2.06 | 1 | 100 | 0 | 0 | 19.72 | 1 | 1 |
| 3 | 1.974 | 1 | 100 | 0 | 0 | 22.243 | 1 | 1 |

/, QPlogS, QPLogKhsa, QPlogBB, #metabol.

| #ringatom | #in34 | #in56 | #noncon | #nonHatm | RuleOfThr | Jm |
|---|---|---|---|---|---|---|
| 16 | 0 | 16 | 5 | 25 | 0 | 0.188 |
| 14 | 0 | 14 | 7 | 22 | 0 | 0.08 |
| 14 | 0 | 14 | 7 | 22 | 0 | 0.053 |
| 14 | 0 | 14 | 7 | 21 | 0 | 0.056 |
| 10 | 0 | 10 | 0 | 14 | 0 | 6.263 |
| 6 | 0 | 6 | 0 | 12 | 0 | 1.306 |
| 16 | 0 | 16 | 0 | 21 | 0 | 0.006 |
| 16 | 0 | 16 | 0 | 22 | 1 | 0.002 |
| 10 | 0 | 10 | 0 | 13 | 0 | 81.26 |
| 18 | 14 | 4 | 12 | 23 | 1 | 0.001 |
| 22 | 0 | 22 | 20 | 31 | 1 | 0 |
| 17 | 0 | 17 | 15 | 30 | 1 | 0 |
