# Supplementary material for: Protective Mechanisms of Nootropic Herb Shankhpushpi (Convolvulus pluricaulis) against Dementia: Network Pharmacology and Computational Approach
Source: Evid Based Complement Alternat Med. 2022 Oct 3;2022:1015310. doi: 10.1155/2022/1015310 (PMC9550454; doi:10.1155/2022/1015310)
Supplement: Supplementary Materials — Supplementary Figure S1. Protein-protein interaction network. Node size is proportional to its degree of connectivity. Supplementary Table S1. Target genes for molecular docking. Supplementary Table S2. QikProp ADMET of CP compounds. Supplementary Table S3. Targets for CP compounds. Supplementary Table S4 data for CP compound-target-disease network. Supplementary Table S5. MM-GBSA free energy calculation result. [file 1015310.f1.zip › Suppl-Table S3-1.pdf]

Compound-Target_network data

Compounds	Related target
Scopoletin	Prostaglandin G/H synthase 1
Scopoletin	Ribosyldihydronicotinamide dehydrogenase [quinone]
Scopoletin	Prostaglandin G/H synthase 2
Scopoletin	Carbonic anhydrase 1
Scopoletin	Beta-2 adrenergic receptor
Scopoletin	Leukotriene A-4 hydrolase
Scopoletin	Amine oxidase [flavin-containing] B
Scopoletin	Glutamate receptor 2
Scopoletin	cAMP-dependent protein kinase catalytic subunit alpha
Scopoletin	Sodium-dependent noradrenaline transporter
Scopoletin	Queuine tRNA-ribosyltransferase
4-Hydroxycinnamic acid	Prostaglandin G/H synthase 1
4-Hydroxycinnamic acid	Prostaglandin G/H synthase 2
4-Hydroxycinnamic acid	Amine oxidase [flavin-containing] B
4-Hydroxycinnamic acid	Amine oxidase [flavin-containing] A
4-Hydroxycinnamic acid	Lysozyme
4-Hydroxycinnamic acid	Chymotrypsinogen B
4-Hydroxycinnamic acid	Group IIE secretory phospholipase A2
4-Hydroxycinnamic acid	Nitric oxide synthase, endothelial
4-Hydroxycinnamic acid	Tyrosinase
4-Hydroxycinnamic acid	C-C motif chemokine 16
Kaempferol	Nitric oxide synthase, inducible
Kaempferol	Prostaglandin G/H synthase 1
Kaempferol	Androgen receptor
Kaempferol	Peroxisome proliferator-activated receptor gamma
Kaempferol	Prostaglandin G/H synthase 2
Kaempferol	Heat shock protein HSP 90-alpha
Kaempferol	Phosphatidylinositol-4,5-bisphosphate 3-kinase catalytic subunit
Kaempferol	cAMP-dependent protein kinase catalytic subunit alpha
Kaempferol	Nuclear receptor coactivator 2
Kaempferol	Trypsin-1
Kaempferol	Progesterone receptor
Kaempferol	Muscarinic acetylcholine receptor M1
Kaempferol	Nitric-oxide synthase, endothelial
Kaempferol	Gamma-aminobutyric-acid receptor subunit alpha-2
Kaempferol	Acetylcholinesterase
Kaempferol	Sodium-dependent noradrenaline transporter
Kaempferol	Muscarinic acetylcholine receptor M2
Kaempferol	Alpha-1B adrenergic receptor
Kaempferol	Gamma-aminobutyric-acid receptor subunit alpha-1
Kaempferol	DNA topoisomerase 2-alpha

Kaempferol	Coagulation factor VII
Kaempferol	Calmodulin
Kaempferol	Transcription factor p65
Kaempferol	Inhibitor of nuclear factor kappa-B kinase subunit beta
Kaempferol	RAC-alpha serine/threonine-protein kinase
Kaempferol	Apoptosis regulator Bcl-2
Kaempferol	Apoptosis regulator BAX
Kaempferol	Tumor necrosis factor
Kaempferol	Transcription factor AP-1
Kaempferol	Activator of 90 kDa heat shock protein ATPase homolog 1
Kaempferol	Caspase-3
Kaempferol	Mitogen-activated protein kinase 8
Kaempferol	Xanthine dehydrogenase/oxidase
Kaempferol	Interstitial collagenase
Kaempferol	Signal transducer and activator of transcription 1-alpha/beta
Kaempferol	Cell division control protein 2 homolog
Kaempferol	Heme oxygenase 1
Kaempferol	Cytochrome P450 3A4
Kaempferol	Cytochrome P450 1A1
Kaempferol	Intercellular adhesion molecule 1
Kaempferol	E-selectin
Kaempferol	Vascular cell adhesion protein 1
Kaempferol	Nuclear receptor subfamily 1 group I member 2
Kaempferol	Cytochrome P450 1B1
Kaempferol	Arachidonate 5-lipoxygenase
Kaempferol	Hyaluronan synthase 2
Kaempferol	Aryl hydrocarbon receptor
Kaempferol	26S proteasome non-ATPase regulatory subunit 3
Kaempferol	Solute carrier family 2, facilitated glucose transporter member 4
Kaempferol	Nuclear receptor subfamily 1 group I member 3
Kaempferol	Insulin receptor
Kaempferol	Type I iodothyronine deiodinase
Kaempferol	Serine/threonine-protein phosphatase 2B catalytic subunit alpha
Kaempferol	Peroxidase C1A
Kaempferol	Glutathione S-transferase Mu 1
Kaempferol	Glutathione S-transferase Mu 2
Kaempferol	Aldo-keto reductase family 1 member C3
Kaempferol	Antileukoproteinase
Quercetin	Prostaglandin G/H synthase 1
Quercetin	Androgen receptor
Quercetin	Peroxisome proliferator-activated receptor gamma
Quercetin	Prostaglandin G/H synthase 2
Quercetin	Heat shock protein HSP 90-alpha

Quercetin	Phosphatidylinositol-4,5-bisphosphate 3-kinase catalytic subunit
Quercetin	Nuclear receptor coactivator 2
Quercetin	Aldose reductase
Quercetin	Trypsin-1
Quercetin	DNA topoisomerase 2-alpha
Quercetin	Potassium voltage-gated channel subfamily H member 2
Quercetin	Sodium channel protein type 5 subunit alpha
Quercetin	Coagulation factor X
Quercetin	Beta-2 adrenergic receptor
Quercetin	Stromelysin-1
Quercetin	cAMP-dependent protein kinase catalytic subunit alpha
Quercetin	Coagulation factor VII
Quercetin	Nitric-oxide synthase, endothelial
Quercetin	Retinoic acid receptor RXR-alpha
Quercetin	Acetylcholinesterase
Quercetin	Gamma-aminobutyric-acid receptor subunit alpha-1
Quercetin	Amine oxidase [flavin-containing] B
Quercetin	Transcription factor p65
Quercetin	Epidermal growth factor receptor
Quercetin	RAC-alpha serine/threonine-protein kinase
Quercetin	G1/S-specific cyclin-D1
Quercetin	Apoptosis regulator Bcl-2
Quercetin	Bcl-2-like protein 1
Quercetin	Proto-oncogene c-Fos
Quercetin	Cyclin-dependent kinase inhibitor 1
Quercetin	Eukaryotic translation initiation factor 6
Quercetin	Apoptosis regulator BAX
Quercetin	Caspase-9
Quercetin	Urokinase-type plasminogen activator
Quercetin	72 kDa type IV collagenase
Quercetin	Matrix metalloproteinase-9
Quercetin	Mitogen-activated protein kinase 1
Quercetin	Interleukin-10
Quercetin	Retinoblastoma-associated protein
Quercetin	Tumor necrosis factor
Quercetin	Transcription factor AP-1
Quercetin	Interleukin-6
Quercetin	Cyclin-dependent kinase inhibitor 2A, isoforms 1/2/3
Quercetin	Activator of 90 kDa heat shock protein ATPase homolog 1
Quercetin	Caspase-3
Quercetin	Cellular tumor antigen p53
Quercetin	ETS domain-containing protein Elk-1
Quercetin	NF-kappa-B inhibitor alpha
Quercetin	Ornithine decarboxylase

Quercetin	Xanthine dehydrogenase/oxidase
Quercetin	Caspase-8
Quercetin	DNA topoisomerase 1
Quercetin	RAF proto-oncogene serine/threonine-protein kinase
Quercetin	Superoxide dismutase [Cu-Zn]
Quercetin	Protein kinase C alpha type
Quercetin	Interstitial collagenase
Quercetin	Hypoxia-inducible factor 1-alpha
Quercetin	Signal transducer and activator of transcription 1-alpha/beta
Quercetin	Protein CBFA2T1
Quercetin	Probable E3 ubiquitin-protein ligase HERC5
Quercetin	Cell division control protein 2 homolog
Quercetin	78 kDa glucose-regulated protein
Quercetin	Receptor tyrosine-protein kinase erbB-2
Quercetin	Acetyl-CoA carboxylase 1
Quercetin	Heme oxygenase 1
Quercetin	Cytochrome P450 3A4
Quercetin	Caveolin-1
Quercetin	Myc proto-oncogene protein
Quercetin	Tissue factor
Quercetin	Gap junction alpha-1 protein
Quercetin	Cytochrome P450 1A1
Quercetin	Intercellular adhesion molecule 1
Quercetin	Interleukin-1 beta
Quercetin	C-C motif chemokine ligand 2
Quercetin	E-selectin
Quercetin	Vascular cell adhesion protein 1
Quercetin	Prostaglandin E2 receptor, EP3 subtype
Quercetin	Interleukin-8
Quercetin	Protein kinase C beta type
Quercetin	Baculoviral IAP repeat-containing protein 5
Quercetin	Dual oxidase 2
Quercetin	Nitric oxide synthase, endothelial
Quercetin	Heat shock protein beta-1
Quercetin	Transforming growth factor beta-1
Quercetin	Maltase-glucoamylase, intestinal
Quercetin	Interleukin-2
Quercetin	Nuclear receptor subfamily 1 group I member 2
Quercetin	Cytochrome P450 1B1
Quercetin	G2/mitotic-specific cyclin-B1
Quercetin	Tissue-type plasminogen activator
Quercetin	Thrombomodulin
Quercetin	Plasminogen activator inhibitor 1

Quercetin	Interferon gamma
Quercetin	Arachidonate 5-lipoxygenase
Quercetin	Phosphatidylinositol-3,4,5-trisphosphate 3-phosphatase and dual
Quercetin	Interleukin-1 alpha
Quercetin	Myeloperoxidase
Quercetin	DNA topoisomerase 2-alpha
Quercetin	Neutrophil cytosol factor 1
Quercetin	ATP-binding cassette sub-family G member 2
Quercetin	Hyaluronan synthase 2
Quercetin	Nuclear factor erythroid 2-related factor 2
Quercetin	NAD(P)H dehydrogenase [quinone] 1
Quercetin	Poly [ADP-ribose] polymerase 1
Quercetin	Aryl hydrocarbon receptor
Quercetin	26S proteasome non-ATPase regulatory subunit 3
Quercetin	Solute carrier family 2, facilitated glucose transporter member 4
Quercetin	Collagen alpha-1(III) chain
Quercetin	DNA gyrase subunit B
Quercetin	C-X-C motif chemokine 11
Quercetin	C-X-C motif chemokine 2
Quercetin	DDB1- and CUL4-associated factor 5
Quercetin	Nuclear receptor subfamily 1 group I member 3
Quercetin	Serine/threonine-protein kinase Chk2
Quercetin	Insulin receptor
Quercetin	Claudin-4
Quercetin	Peroxisome proliferator-activated receptor alpha
Quercetin	Peroxisome proliferator-activated receptor delta
Quercetin	Heat shock factor protein 1
Quercetin	C-reactive protein
Quercetin	C-X-C motif chemokine 10
Quercetin	Inhibitor of nuclear factor kappa-B kinase subunit alpha
Quercetin	Osteopontin
Quercetin	Runt-related transcription factor 2
Quercetin	Ras association domain-containing protein 1
Quercetin	Transcription factor E2F1
Quercetin	Transcription factor E2F2
Quercetin	Prostatic acid phosphatase
Quercetin	Cathepsin D
Quercetin	Insulin-like growth factor-binding protein 3
Quercetin	Insulin-like growth factor II
Quercetin	CD40 ligand
Quercetin	Interferon regulatory factor 1
Quercetin	Receptor tyrosine-protein kinase erbB-3
Quercetin	Serum paraoxonase/arylesterase 1

Quercetin	Type I iodothyronine deiodinase
Quercetin	Procollagen C-endopeptidase enhancer 1
Quercetin	Puromycin-sensitive aminopeptidase
Quercetin	Hexokinase-2
Quercetin	Homeobox protein Nkx-3.1
Quercetin	Ras GTPase-activating protein 1
Quercetin	Peroxidase C1A
Quercetin	Glutathione S-transferase Mu 1
Quercetin	Glutathione S-transferase Mu 2
Ayapanin	Prostaglandin G/H synthase 1
Ayapanin	Prostaglandin G/H synthase 2
Ayapanin	Alpha-2A adrenergic receptor
Ayapanin	Sodium-dependent dopamine transporter
Ayapanin	Beta-2 adrenergic receptor
Ayapanin	Sodium-dependent serotonin transporter
Ayapanin	Leukotriene A-4 hydrolase
Ayapanin	Amine oxidase [flavin-containing] B
Ayapanin	Amine oxidase [flavin-containing] A
Ayapanin	cAMP-dependent protein kinase catalytic subunit alpha
Ayapanin	Muscarinic acetylcholine receptor M1
Ayapanin	Alpha-1A adrenergic receptor
Ayapanin	cAMP-dependent protein kinase inhibitor alpha

Gene
PTGS1
NQO2
PTGS2
CA1
ADRB2
LTA4H
MAOB
GRIA2
PRKACA
SLC6A2
QTRT1
PTGS1
PTGS2
MAOB
MAOA
LYZ
CTRB
PLA2G2E
NOS3
TYR
CCL16
NOS2
PTGS1
AR
PPARG
PTGS2
HSP90AA1
PIK3CG
PRKACA
NCOA2
PRSS1
PGR
CHRM1
NOS3
GABRA2
ACHE
SLC6A2
CHRM2
ADRA1B
GABRA1
TOP2A

F7
CALM
RELA
IKBKB
AKT1
BCL2
BAX
TNF
JUN
AHSA1
CASP3
MAPK8
XDH
MMP1
STAT1
CDC2
HMOX1
CYP3A4
CYP1A1
ICAM1
SELE
VCAM1
NR1I2
CYP1B1
ALOX5
HAS2
AHR
PSMD3
SLC2A4
NR1I3
INSR
DIO1
PPP3CA
PRXC1A
GSTM1
GSTM2
AKR1C3
SLPI
PTGS1
AR
PPARG
PTGS2
HSP90AA1

PIK3CG
NCOA2
AKR1B1
PRSS1
TOP2A
KCNH2
SCN5A
F10
ADRB2
MMP3
PRKACA
F7
NOS3
RXRA
ACHE
GABRA1
MAO-B
RELA
EGFR
AKT1
CCND1
BCL2
BCL2L1
FOS
CDKN1A
EIF6
BAX
CASP9
PLAU
MMP2
MMP9
MAPK1
IL10
RB1
TNF
JUN
IL6
CDKN2A
AHSA1
CASP3
TP53
ELK1
NFKBIA
ODC1

XDH
CASP8
TOP1
RAF1
SOD1
PRKCA
MMP1
HIF1A
STAT1
RUNX1T1
HERC5
CDC2
GRP78
ERBB2
ACACA
HMOX1
CYP3A4
CAV1
MYC
F3
GJA1
CYP1A1
ICAM1
IL1B
CCL2
SELE
VCAM1
PTGER3
CXCL8
PRKCB
BIRC5
DUOX2
NOS3
HSPB1
TGFB1
MGAM
IL2
NR1I2
CYP1B1
CCNB1
PLAT
THBD
SERPINE1

IFNG
ALOX5
PTEN
IL1A
MPO
TOP2A
NCF1
ABCG2
HAS2
NFE2L2
NQO1
PARP1
AHR
PSMD3
SLC2A4
COL3A1
GYRB
CXCL11
CXCL2
DCAF5
NR1I3
CHEK2
INSR
CLDN4
PPARA
PPARD
HSF1
CRP
CXCL10
CHUK
SPP1
RUNX2
RASSF1
E2F1
E2F2
ACP3
CTSD
IGFBP3
IGF2
CD40LG
IRF1
ERBB3
PON1

DIO1
PCOLCE
NPEPPS
HK2
NKX3-1
RASA1
PRXC1A
GSTM1
GSTM2
PTGS1
PTGS2
ADRA2A
SLC6A3
ADRB2
SLC6A4
LTA4H
MAOB
MAOA
PRKACA
CHRM1
ADRA1A
PKIA
