# Supplementary material for: Protective Mechanisms of Nootropic Herb Shankhpushpi (Convolvulus pluricaulis) against Dementia: Network Pharmacology and Computational Approach
Source: Evid Based Complement Alternat Med. 2022 Oct 3;2022:1015310. doi: 10.1155/2022/1015310 (PMC9550454; doi:10.1155/2022/1015310)
Supplement: Supplementary Materials — Supplementary Figure S1. Protein-protein interaction network. Node size is proportional to its degree of connectivity. Supplementary Table S1. Target genes for molecular docking. Supplementary Table S2. QikProp ADMET of CP compounds. Supplementary Table S3. Targets for CP compounds. Supplementary Table S4 data for CP compound-target-disease network. Supplementary Table S5. MM-GBSA free energy calculation result. [file 1015310.f1.zip › Suppl-Table S3-2.pdf]

Targets for CP compounds

PTGS1
PTGS2
ADRA2A
SLC6A3
ADRB2
SLC6A4
LTA4H
MAOB
MAOA
PRKACA
CHRM1
ADRA1A
DPP4
PKIA
MAOA
LYZ
CTRB
PLA2G2E
NOS3
TYR
CCL16
NOS2
AR
PPARG
HSP90AA1
PIK3CG
NCOA2
PRSS1
PGR
GABRA2
ACHE
SLC6A2
CHRM2
ADRA1B
GABRA1
TOP2A
F7
CALM
RELA
IKBKB
AKT1
BCL2

BAX
TNF
JUN
AHSA1
CASP3
MAPK8
XDH
MMP1
STAT1
CDC2
HMOX1
CYP3A4
CYP1A1
ICAM1
SELE
VCAM1
NR1I2
CYP1B1
ALOX5
HAS2
AHR
PSMD3
SLC2A4
NR1I3
INSR
DIO1
PPP3CA
PRXC1A
GSTM1
GSTM2
AKR1C3
SLPI
AKR1B1
0
KCNH2
SCN5A
F10
MMP3
RXRA
MAO-B
EGFR
CCND1
BCL2L1
FOS

CDKN1A
EIF6
CASP9
PLAU
MMP2
MMP9
MAPK1
IL10
RB1
IL6
CDKN2A
TP53
ELK1
NFKBIA
ODC1
CASP8
TOP1
RAF1
SOD1
PRKCA
HIF1A
RUNX1T1
HERC5
GRP78
ERBB2
ACACA
CAV1
MYC
F3
GJA1
IL1B
CCL2
PTGER3
CXCL8
PRKCB
BIRC5
DUOX2
HSPB1
TGFB1
MGAM
IL2
CCNB1
PLAT

THBD
SERPINE1
IFNG
PTEN
IL1A
MPO
NCF1
ABCG2
NFE2L2
NQO1
PARP1
COL3A1
GYRB
CXCL11
CXCL2
DCAF5
CHEK2
CLDN4
PPARA
PPARD
HSF1
CRP
CXCL10
CHUK
SPP1
RUNX2
RASSF1
E2F1
E2F2
ACP3
CTSD
IGFBP3
IGF2
CD40LG
IRF1
ERBB3
PON1
PCOLCE
NPEPPS
HK2
NKX3-1
RASA1
NQO2

CA1
GRIA2
QTRT1
