# Supplementary material for: Protective Mechanisms of Nootropic Herb Shankhpushpi (Convolvulus pluricaulis) against Dementia: Network Pharmacology and Computational Approach
Source: Evid Based Complement Alternat Med. 2022 Oct 3;2022:1015310. doi: 10.1155/2022/1015310 (PMC9550454; doi:10.1155/2022/1015310)
Supplement: Supplementary Materials — Supplementary Figure S1. Protein-protein interaction network. Node size is proportional to its degree of connectivity. Supplementary Table S1. Target genes for molecular docking. Supplementary Table S2. QikProp ADMET of CP compounds. Supplementary Table S3. Targets for CP compounds. Supplementary Table S4 data for CP compound-target-disease network. Supplementary Table S5. MM-GBSA free energy calculation result. [file 1015310.f1.zip › Suppl-Table S4.pdf]

Table S4_data for CP compound-target-disease network

CP compounds' Targets	AD-associated target Genes	PD-associated target Genes
ABCG2	*ABCG2*	*ABCG2*
ACACA	*ACACA*	*ACACA*
ACHE	*ACHE*	*ACHE*
ACP3	*ADRA1A*	*ACP3*
ADRA1A	*ADRA2A*	*ADRB2*
ADRA1B	*ADRB2*	*AHR*
ADRA2A	*AHR*	*AHR*
ADRB2	*AHSA1*	*AHSA1*
AHR	*AKT1*	*AKT1*
AHSA1	*ALOX5*	*ALOX5*
AKR1B10	*AR*	*AR*
AKR1C3	*BAX*	*BAX*
AKT1	*BCL2*	*BCL2*
ALOX5	*CASP3*	*BCL2L1*
AR	*CASP8*	*CASP3*
BAX	*CASP9*	*CASP8*
BCL2	*CAV1*	*CASP9*
BCL2L1	*CCL2*	*CAV1*
BIRC5	*CD40LG*	*CCL2*
CA1	*CDKN1A*	*CDKN1A*
CALM	*CDKN2A*	*CDKN2A*
CASP3	*CHRM1*	*COL3A1*
CASP8	*CHRM2*	*CP*
CASP9	*COL3A1*	*CRP*
CAV1	*CP*	*CTSD*
CCL16	*CRP*	*CXCL11*
CCL2	*CTSD*	*CXCL2*
CCNB1	*CXCL10*	*CXCL8*
CCND1	*CXCL11*	*CYP1A1*
CD40LG	*CXCL2*	*CYP1B1*
CDC2	*CXCL8*	*CYP3A4*
CDKN1A	*CYP3A4*	*DCAF5*
CDKN2A	*E2F1*	*E2F1*
CHEK2	*EGFR*	*EGFR*
CHRM1	*ELK1*	*ERBB2*
CHRM2	*ERBB2*	*ERBB3*
CHUK	*F3*	*GRIA2*
CLDN4	*FOS*	*GSTM1*
COL3A1	*GABRA2*	*GSTM2*
CRP	*GJA1*	*GYRB*
CTRB	*GRIA2*	*HAS2*

CTSD	*GSTM1*	*HAS2*
CXCL10	*GSTM2*	*HIF1A*
CXCL11	*HIF1A*	*HK2*
CXCL2	*HMOX1*	*HMOX1*
CXCL8	*HSF1*	*HSF1*
CYP1A1	*HSP90AA1*	*HSP90AA1*
CYP1B1	*HSPB1*	*HSPB1*
CYP3A4	*ICAM1*	*ICAM1*
DCAF5	*IFNG*	*IFNG*
DIO1	*IGF2*	*IGF2*
DUOX2	*IGFBP3*	*IKBKB*
E2F1	*IKBKB*	*IL10*
E2F2	*IL10*	*IL1A*
EGFR	*IL1A*	*IL1B*
EIF6	*IL1B*	*IL6*
ELK1	*IL6*	*INSR*
ERBB2	*INSR*	*MAOA*
ERBB3	*JUN*	*MAOB*
F10	*LYZ*	*MAPK1*
F3	*MAOA*	*MAPK8*
F7	*MAOB*	*MGAM*
FOS	*MAPK1*	*MMP1*
GABRA1	*MAPK8*	*MMP2*
GABRA2	*MGAM*	*MMP9*
GJA1	*MMP1*	*MPO*
GRIA2	*MMP2*	*MYC*
GRP78	*MMP3*	*NCF1*
GSTM1	*MMP9*	*NFE2L2*
GSTM2	*MPO*	*NFE2L2*
GYRB	*MYC*	*NFKBIA*
HAS2	*NCF1*	*NOS2*
HERC5	*NFE2L2*	*NOS3*
HIF1A	*NOS2*	*NPEPPS*
HK2	*NOS3*	*NQO1*
HMOX1	*NPEPPS*	*NQO1*
HSF1	*NQO1*	*NQO2*
HSP90AA1	*NQO2*	*NR1I2*
HSPB1	*NR1I2*	*NR1I3*
ICAM1	*NR1I3*	*NR1I3*
IFNG	*PARP1*	*PARP1*
IGF2	*PGR*	*PARP1*
IGFBP3	*PIK3CG*	*PIK3CG*
IKBKB	*PLAT*	*PON1*

IL10
IL1A
IL1B
IL2
IL6
INSR
IRF1
JUN
KCNH2
LTA4H
LYZ
MAOA
MAOA
MAOB
MAO-B
MAPK1
MAPK8
MGAM
MMP1
MMP2
MMP3
MMP9
MPO
MYC
NCF1
NCOA2
NFE2L2
NFKBIA
NKX3-1
NOS2
NOS3
NPEPPS
NQO1
NQO2
NR1I2
NR1I3
ODC1
PARP1
PCOLCE
PGR
PIK3CG
PKIA
PLA2G2E

PLAU
PON1
PPARA
PPARD
PPARG
PRKACA
PRKCA
PRKCB
PSMD3
PTEN
PTGS1
PTGS2
RAF1
RASA1
RB1
RELA
RUNX1T1
RXRA
SELE
SERPINE1
SLC2A4
SLC6A2
SLC6A3
SLC6A4
SLPI
SOD1
SPP1
STAT1
TGFB1
TNF
TP53
TYR
VCAM1

PPARA
PPARD
PPARG
PRKACA
PSMD3
PTEN
PTGS2
RB1
RXRA
SELE
SERPINE1
SLC2A4
SLC2A4
SLC6A2
SLC6A3
SLC6A4
SOD1
SPP1
STAT1
TGFB1
TNF
TOP2A
TP53
TYR
VCAM1

PLAT
PLAU
PON1
PPARA
PPARD
PPARG
PPP3CA
PRKACA
PRKCA
PRKCB
PRSS1
PRXC1A
PSMD3
PTEN
PTGER3
PTGS1
PTGS2
QTRT1
RAF1
RASA1
RASSF1
RB1
RELA
RUNX1T1
RUNX2
RXRA
SCN5A
SELE
SERPINE1
SLC2A4
SLC6A2
SLC6A3
SLC6A4
SLPI
SOD1
SPP1
STAT1
TGFB1
THBD
TNF
TOP1
TOP2A
TP53

TYR
VCAM1
XDH

Dementia-associated target Genes	Overlaping targets
ACHE	*ACHE*
AHR	*AHR*
AKT1	*AKT1*
BAX	*BAX*
BCL2	*BCL2*
CAV1	*CAV1*
CDKN1A	*CDKN1A*
CHRM1	*CTSD*
CTSD	*CXCL8*
CXCL10	*E2F1*
CXCL8	*EGFR*
E2F1	*HMOX1*
EGFR	*HSF1*
HMOX1	*ICAM1*
HSF1	*IFNG*
ICAM1	*IGF2*
IFNG	*IKBKB*
IGF2	*IL10*
IGFBP3	*IL1A*
IKBKB	*IL1B*
IL10	*IL6*
IL1A	*INSR*
IL1B	*MAOA*
IL6	*MAPK1*
INSR	*MMP2*
MAOA	*MMP9*
MAPK1	*MPO*
MMP2	*NOS2*
MMP3	*NOS3*
MMP9	*NR1I2*
MPO	*PIK3CG*
NOS2	*PPARA*
NOS3	*PPARG*
NR1I2	*PTGS2*
PIK3CG	*SELE*
PLAU	*SERPINE1*
PPARA	*SLC6A3*
PPARG	*SOD1*
PPP3CA	*SPP1*
PRKCB	*STAT1*
PTGS2	*TGFB1*

SELE
SERPINE1
SLC6A3
SOD1
SPP1
STAT1
TGFB1
TNF
TP53
VCAM1
TNF
TP53
VCAM1
