# Supplementary material for: Protective Mechanisms of Nootropic Herb Shankhpushpi (Convolvulus pluricaulis) against Dementia: Network Pharmacology and Computational Approach
Source: Evid Based Complement Alternat Med. 2022 Oct 3;2022:1015310. doi: 10.1155/2022/1015310 (PMC9550454; doi:10.1155/2022/1015310)
Supplement: Supplementary Materials — Supplementary Figure S1. Protein-protein interaction network. Node size is proportional to its degree of connectivity. Supplementary Table S1. Target genes for molecular docking. Supplementary Table S2. QikProp ADMET of CP compounds. Supplementary Table S3. Targets for CP compounds. Supplementary Table S4 data for CP compound-target-disease network. Supplementary Table S5. MM-GBSA free energy calculation result. [file 1015310.f1.zip › Suppl-Table S5.pdf]

Table S5_MM-GBSA free energy calculation result

title	r_psp_MMGBSA_dG_Bind	r_psp_MM	r_psp_MM	r_psp_MM
PTGS2				
Scopoletin	-37.60080127	-24.0079	1.629448	-2.51947
4-Hydroxycinnamic acid	-27.86252475	-13.4741	2.531961	-1.2746
Kaempferol	-28.47589113	-92.8469	1.329701	-4.1141
Quercetin	-46.27227234	-27.3642	0.853187	-2.50654
Ayapanin	-32.98505574	-3.88983	0.316931	-0.00334
NOS3				
Scopoletin	-34.98273163	-34.0881	8.399126	-2.81397
4-Hydroxycinnamic acid	-19.60096707	-17.883	10.10579	-0.9016
Kaempferol	11.51092246	22.33073	3.787311	-2.40728
Quercetin	-16.50613717	-15.9065	8.026233	-2.2576
Ayapanin	-15.62671476	-13.1969	2.740368	-0.54326
PTGS1				
Scopoletin	-36.28116934	-20.1373	2.369418	-2.3742
4-Hydroxycinnamic acid	-30.48016405	-13.4596	-0.2206	-1.00698
Kaempferol	-22.18027117	-14.8471	2.431853	-1.15623
Quercetin	-35.13088847	-20.9816	2.826982	-1.47805
Ayapanin	-27.17363778	-5.70125	-0.04662	-0.63424
INSR				
Scopoletin	29.54110923	-59.8973	6.344893	-2.99235
4-Hydroxycinnamic acid	-21.4633956	-21.7634	5.707014	-1.04307
Kaempferol	25.57890481	-94.0004	4.271868	-0.5289
Quercetin	40.09119365	-48.7411	4.381571	-1.28588
Ayapanin	50.06038707	-32.4943	3.8994	-0.05247
NR1I3				
Scopoletin	-56.01405518	-32.716	3.227228	-0.82442
4-Hydroxycinnamic acid	-43.32734579	-11.5825	3.284657	-0.70903
Kaempferol	-13.70371972	48.40166	0.743822	-0.62105
Quercetin	-49.91616587	-23.3415	6.916772	-0.85534
Ayapanin	-38.30447952	-8.12912	1.918072	-0.34857
NR1I2				
Scopoletin	-39.13494472	-18.7804	1.697287	-2.04615
4-Hydroxycinnamic acid	-35.424107	-8.19464	1.407724	-0.56932
Kaempferol	-18.36887696	-21.1222	3.15267	-1.56746
Quercetin	-34.4923938	-23.0771	1.771791	-1.80474
Ayapanin	-36.3173049	-3.55719	0.953462	-0.40195
HMOX1				
Scopoletin	-33.30092926	-14.8675	1.267954	-1.18965
4-Hydroxycinnamic acid	-36.2681757	-7.21696	4.882745	-1.80341

Kaempferol		-16.96161664	20.48696	4.047998	-0.9091
Quercetin		-34.20019731	-2.44644	0.595982	-1.12486
Ayapanin		-37.80482978	-2.83636	0.345038	-0.01697
ACHE					
Scopoletin		-43.13985184	-35.6414	3.805607	-2.96885
4-Hydroxycinnamic acid		-39.80323709	-16.4301	2.050396	-1.35739
Kaempferol		-6.302466557	30.71241	1.444767	-1.08961
Quercetin		11.42716559	-54.1566	1.454632	-0.97734
Ayapanin		-41.44830429	-11.281	3.996616	-0.57415
PPARG					
Scopoletin		-41.04259001	-18.3711	2.700566	-1.5804
4-Hydroxycinnamic acid		-48.89073968	-20.5122	4.957828	-2.55748
Kaempferol		-23.949427	52.44628	1.345164	-2.70303
Quercetin		-4.977189325	3.410362	7.625514	-2.74501
Ayapanin		-43.36886213	-14.9081	0.568943	-2.571
MAOA					
Scopoletin		-51.18094319	-38.4282	6.524859	-2.599
4-Hydroxycinnamic acid		-28.06330989	-11.2396	2.499148	-0.56372
Kaempferol		-17.13274775	-60.938	1.880354	-2.05508
Quercetin		-13.93043484	-144.824	3.355836	-1.87068
Ayapanin		-34.58920552	-11.8002	-0.37647	-0.81115
MAOB					
Scopoletin		-34.04463499	-25.534	5.074216	-1.81288
4-Hydroxycinnamic acid		-46.57202698	-20.1127	1.10706	-2.04875
Kaempferol		-29.61393996	-17.0671	0.515588	-2.4847
Quercetin		-26.66736055	-21.8723	4.352306	-1.05466
Ayapanin		-38.57804154	-17.3686	0.991407	-0.93545
TRKB					
Scopoletin		-34.67008334	-33.9275	4.074114	-2.97174
4-Hydroxycinnamic acid		-3.064918958	63.77819	0.702328	-1.21878
Kaempferol		-14.75568473	70.67331	1.142954	-0.82127
Quercetin		-8.792047271	50.72672	-0.18959	-1.15546
Ayapanin		-20.71866403	-7.06861	0.071117	-1.18657

r_psp_MM	r_psp_MM	r_psp_MM	r_psp_MMGBSA_d	r_psp_MM	r_psp_MMGBSA_dG_Bind_vdW
-12.5802	-7.02736	0	44.3486788		-37.44395313
-9.95306	-3.59866	0	19.31279528		-21.4068473
-7.40547	-1.93389	0	99.29137362		-22.79656923
-12.5935	-6.99743	0	38.42554806		-36.08935459
-14.1184	-3.17024	0	20.72405917		-32.84424878
-23.784	-10.4461	0	65.40657751		-37.65629699
-24.1046	-13.3664	0	49.28372017		-22.73481333
-15.5275	-3.65979	0	31.28716766		-24.2996607
-23.6625	-10.3682	0	67.23010883		-39.56768177
-21.3063	-10.1892	0	59.21008804		-32.34154844
-9.65533	-2.28028	0	18.52582695		-22.72932153
-10.9741	-1.70067	0	11.56195415		-14.68016944
-10.5853	-0.45011	0	19.36209862		-16.93547914
-12.6633	-1.61408	0	19.81448725		-21.03529683
-11.3242	-2.72482	0	7.603422741		-14.34592063
-8.24639	-0.75099	0	122.6901839		-27.60691643
-9.48269	-0.05021	0	26.2782369		-21.1092609
-4.76638	-0.0025	0	133.8469529		-13.24177624
-6.52768	-0.17221	0	125.8359122		-33.39942261
-9.03666	-1.08285	0	113.0689399		-24.241687
-17.8519	-8.46615	0	32.39517505		-31.77803757
-15.0666	-3.08682	0	12.0994791		-28.26655045
-13.4935	-0.82168	0	-23.48784178		-24.42515986
-18.1787	-8.45282	0	26.94016599		-32.94468973
-14.4521	-6.83483	0	13.40035291		-23.85830412
-10.5545	-2.34245	0	27.94684364		-35.05560552
-14.0343	-5.0438	0	16.29928604		-25.28905552
-9.48866	-0.71943	0	31.82783355		-20.45159766
-11.4242	-1.51082	0	28.95859993		-27.4058992
-15.8026	-7.38885	0	13.07051233		-23.19069921
-12.4788	-1.69268	0	29.34487256		-33.68506656
-13.1706	-1.45092	0	10.36993301		-27.87894804

-11.3937	-3.36164	0	-0.708400912	-25.12377167
-13.042	-4.76816	0	21.09048933	-34.50522381
-17.2554	-1.33342	0	9.958617473	-26.66638224
-16.679	-9.41733	0	53.91635053	-36.15516703
-17.9412	-3.89003	0	18.97489982	-21.2097675
-14.7908	-2.11719	0	2.292442638	-22.75447643
-16.0645	-7.28101	0	118.8769428	-30.42498522
-18.0383	-6.26889	0	15.56259203	-24.84516842
-11.193	-0.00042	0	23.52328067	-36.12146719
-17.0593	-0.11744	0	19.61704645	-33.21912139
-15.1877	-6.85E-08	0	-34.30544776	-25.54464384
-11.7097	-0.06091	0	35.37522453	-36.87268002
-16.1555	-0.11111	0	19.47740798	-29.66952654
-17.4155	-4.64806	0	38.4548227	-33.06986119
-14.107	-3.06263	0	26.72262858	-28.3121719
-8.14396	-0.24721	0	71.13610259	-18.76496576
-14.2025	-4.56776	0	180.6300086	-32.45104783
-12.9757	-2.36691	0	15.17812913	-21.43689664
-19.087	-7.1224	0	48.75566116	-34.31828995
-15.0389	-2.24718	0	19.86549234	-28.09707433
-12.0517	-0.78364	0	33.41922975	-31.16164865
-19.0302	-7.47096	0	51.55572713	-33.1473081
-15.4925	-5.8915	0	28.20279299	-28.08410154
-8.10554	-1.57066	0	25.16540229	-17.33413994
-2.9088	-0.66417	0	-46.16013787	-16.59354906
-8.6541	-1.42434	0	-59.65635821	-16.01588134
-6.27101	-1.61373	0	-32.59890602	-17.69006388
-6.43579	-0.69238	0	11.53438437	-16.94081014

r_psp_Lig_	r_psp_Lig_	r_psp_Lig_	r_psp_Lig_	r_psp_Lig_	r_psp_Lig_	r_psp_Lig_	r_psp_Lig_	r_psp_Lig_
3.593697	-0.27072	1.629448	0	0.03662	0	0	0.271793	
3.889341	-0.43774	2.531961	0	-0.00988	0	0	0.184856	
1.123276	-0.33775	1.329701	0	-0.00194	0	0	0.079071	
2.44133	0.071161	0.853187	0	0.018576	0	0	0.135665	
0.391154	-0.06883	0.316931	0	-0.0015	0	0	0.069784	
8.421655	-1.29888	8.401046	0	-0.17358	0	0	0.858229	
13.04285	-0.44827	10.10771	0	0.021454	0	0	0.329626	
3.835022	-0.04854	3.789231	0	0.001786	0	0	-0.07211	
7.053094	-2.12583	8.028153	0	-0.14385	0	0	1.504379	
2.204665	-0.20509	2.742288	0	-0.01194	0	0	0.048771	
1.750047	0.151381	2.369418	0	-0.0093	0	0	-0.51659	
0.472665	-1.66196	-0.2206	0	0.04707	0	0	0.376863	
2.106219	-0.42543	2.431853	0	-0.00375	0	0	0.100256	
2.039645	-0.11469	2.826982	0	-0.02407	0	0	-0.10034	
0.312327	-0.00207	-0.04662	0	-0.00122	0	0	-0.00771	
7.860242	-1.54875	6.344893	0	0.035552	0	0	0.560681	
2.810139	-2.83333	5.707014	0	0.008656	0	0	1.267137	
3.74693	-0.29231	4.271868	0	-0.00393	0	0	0.059935	
8.374856	11.16077	4.381571	0	0.00843	0	0	-5.59037	
2.866429	-0.32994	3.8994	0	-0.02269	0	0	0.15894	
-1.39131	-12.9253	3.227228	0	-0.00124	0	0	5.430601	
2.627443	-1.02984	3.284657	0	-0.003	0	0	0.407397	
0.70904	-0.00696	0.743822	0	0.002781	0	0	0.159564	
6.042939	-1.65355	6.916772	0	0.016431	0	0	0.176006	
1.833748	-0.13498	1.918072	0	-0.00815	0	0	0.071906	
2.781416	1.021228	1.697287	0	0.04442	0	0	-0.39869	
2.242551	0.801287	1.407724	0	-0.00261	0	0	-0.20507	
2.151454	-0.22632	3.15267	0	-0.01481	0	0	-0.0043	
2.624315	-0.3859	1.771791	0	0.005566	0	0	-0.06337	
1.021946	-0.17215	0.953462	0	-0.00939	0	0	0.030991	
1.79695	-0.73148	1.267954	0	0.012302	0	0	0.232677	
3.819964	0.186719	4.882745	0	0.010976	0	0	0.334859	

3.686067	-0.24101	4.047998	0	-0.00012	0	0	-0.07721
1.517634	-0.52221	0.595982	0	0.017266	0	0	0.136673
0.44195	-0.05821	0.345038	0	0.001004	0	0	0.032433
5.893616	1.33288	3.805607	0	-0.02804	0	0	-0.79114
1.709129	1.131472	2.050396	0	-0.04466	0	0	-0.34352
0.704432	-0.13474	1.444767	0	-0.00163	0	0	-0.13198
3.295254	-0.66673	1.454632	0	0.017594	0	0	0.692627
2.238664	-0.39762	3.996616	0	-0.02235	0	0	-0.14563
1.958042	0.370501	2.700566	0	-0.02645	0	0	0.150584
4.118316	0.062415	4.957828	0	0.022369	0	0	0.423004
2.469413	0.148929	1.345164	0	-0.00068	0	0	-0.01775
14.68475	9.59402	7.625514	0	0.00937	0	0	-3.50904
0.583353	-0.02223	0.568943	0	-0.00022	0	0	-0.01154
7.544325	-0.66253	6.524859	0	0.00495	0	0	-0.22628
1.877313	-0.46649	2.499148	0	-0.01868	0	0	0.135036
1.311722	0.307338	1.880354	0	0.002389	0	0	-0.16574
4.593497	-0.59073	3.355836	0	0.003707	0	0	0.271318
0.439279	0.008972	-0.37647	0	-0.00692	0	0	-0.02658
6.580797	-0.48576	5.074216	0	-0.00478	0	0	0.067606
1.080078	-0.31303	1.10706	0	0.000197	0	0	0.057241
0.759903	-0.01532	0.515588	0	0.005655	0	0	-0.11787
5.911443	-0.17508	4.352306	0	-0.00162	0	0	-0.25293
0.580854	0.012615	0.991407	0	-0.00542	0	0	-0.39133
5.892353	0.044077	4.074114	0	0.019195	0	0	-0.81269
0.570747	-0.15078	0.702328	0	-0.0009	0	0	0.071971
0.535588	-0.10883	1.142954	0	-0.00425	0	0	0.017565
1.413177	0.075765	-0.18959	0	0.01033	0	0	0.133006
0.369962	0.025466	0.071117	0	-0.00134	0	0	0.003957

r_psp_Lig_	r_psp_Liga	r_psp_Liga	r_psp_Liga	r_psp_Liga	r_psp_Liga	r_psp_Liga	r_psp_Liga	r_psp_Liga
1.926554	-72.832	-88.6836	4.859055	0	-1.57907	0	0	-16.4495
1.620146	-3.4652	-21.2911	8.781064	0	-0.87919	0	0	-8.70711
0.054199	-40.1272	-23.3575	3.637854	0	-0.44432	0	0	-29.9182
1.362741	-93.2695	-108.753	4.193919	0	-1.58484	0	0	-14.7804
0.074768	-21.0591	-41.3902	8.37407	0	-0.79794	0	0	-5.86479
0.634846	-71.005	-88.0554	5.829505	0	-1.5778	0	0	-16.0871
3.032326	-6.74481	-26.4567	8.882972	0	-0.87859	0	0	-7.04816
0.164656	-40.1686	-23.458	3.638036	0	-0.44438	0	0	-29.8588
-0.20976	-97.7838	-115.177	2.10862	0	-1.57529	0	0	-11.4671
-0.36937	-21.0867	-41.3898	8.375472	0	-0.79793	0	0	-5.8926
-0.24487	-73.773	-89.4568	3.419707	0	-1.57007	0	0	-14.8351
1.93129	-2.18903	-24.0206	13.15326	0	-0.85297	0	0	-7.67943
0.003291	-40.1296	-23.4582	3.638364	0	-0.44435	0	0	-29.8198
-0.54824	-97.8255	-115.173	2.121094	0	-1.57541	0	0	-11.5084
0.369956	-21.0853	-41.3897	8.374927	0	-0.79794	0	0	-5.89114
2.467866	-69.1218	-81.5581	5.581091	0	-1.58064	0	0	-19.2779
-1.33934	-3.57465	-21.2899	8.780565	0	-0.87919	0	0	-8.81742
-0.28863	-40.0659	-23.3589	3.639567	0	-0.44434	0	0	-29.8554
-1.58554	-94.0183	-112.056	4.277609	0	-1.58467	0	0	-12.3104
-0.83929	-21.1414	-41.3895	8.375282	0	-0.79793	0	0	-5.94733
2.877413	-66.2525	-77.8257	6.550317	0	-1.57976	0	0	-20.8975
-0.03176	-3.57569	-21.29	8.78227	0	-0.87918	0	0	-8.81898
-0.19016	-40.2342	-23.3583	3.638481	0	-0.44433	0	0	-30.0241
0.587281	-93.3596	-107.8	2.934128	0	-1.57722	0	0	-14.0799
-0.0131	-21.1048	-41.3897	8.374908	0	-0.79794	0	0	-5.91072
0.417168	-71.6164	-87.277	4.630893	0	-1.56965	0	0	-15.7637
0.241218	-6.80507	-26.4547	8.88221	0	-0.87859	0	0	-7.10846
-0.75578	-40.128	-23.4585	3.639798	0	-0.44438	0	0	-29.8186
1.296233	-88.8996	-99.963	4.929884	0	-1.58719	0	0	-18.538
0.219023	-20.7893	-41.4269	8.410461	0	-0.79761	0	0	-5.54202
1.015501	-70.7623	-83.8226	3.550148	0	-1.57051	0	0	-17.2185
-1.59534	-6.78081	-26.4559	8.882673	0	-0.87859	0	0	-7.08418

-0.04359	-40.0247	-23.4584	3.636875	0	-0.44437	0	0	-29.7136
1.289927	-97.7591	-115.177	2.116178	0	-1.57533	0	0	-11.4425
0.121681	-20.8463	-41.4265	8.409915	0	-0.79761	0	0	-5.59927
1.574309	-66.4574	-79.0542	6.680449	0	-1.58004	0	0	-19.9402
-1.08456	-2.88852	-25.6282	11.73404	0	-0.80451	0	0	-7.08414
-0.47199	-39.9421	-23.3585	3.637273	0	-0.44432	0	0	-29.731
1.797127	-98.8983	-78.5727	5.080002	0	-0.82022	0	0	-50.9821
-1.19235	-20.7913	-41.4269	8.410051	0	-0.79762	0	0	-5.544
-1.23716	-74.8498	-91.0131	3.5786	0	-1.57019	0	0	-14.4814
-1.3473	-6.84418	-26.4549	8.882944	0	-0.87859	0	0	-7.14804
0.993748	-40.1608	-23.3576	3.638047	0	-0.44432	0	0	-29.9512
0.96489	-105.574	-91.3286	4.313658	0	-0.8196	0	0	-45.8348
0.048398	-20.7988	-41.4266	8.410396	0	-0.79761	0	0	-5.55161
1.90333	-65.2488	-74.5121	5.434823	0	-1.57987	0	0	-22.0583
-0.2717	-3.62155	-21.2899	8.781651	0	-0.87917	0	0	-8.86454
-0.71262	-40.1897	-23.4582	3.639655	0	-0.44439	0	0	-29.8798
1.553361	-98.6199	-78.5751	5.077234	0	-0.82024	0	0	-50.703
0.84028	-21.0272	-41.3903	8.375581	0	-0.79793	0	0	-5.83273
1.929521	-67.8468	-78.7019	5.592672	0	-1.58164	0	0	-20.7777
0.228613	-3.58568	-21.2898	8.780498	0	-0.87919	0	0	-8.82842
0.371848	-40.0709	-23.3579	3.636484	0	-0.44431	0	0	-29.8602
1.988765	-87.9816	-98.4472	4.932465	0	-1.5873	0	0	-19.1236
-0.02643	-20.7709	-41.4269	8.411352	0	-0.79761	0	0	-5.5236
2.567658	-71.5997	-87.1419	4.660799	0	-1.57058	0	0	-15.8847
-0.05187	-36.5224	-23.9587	8.627959	0	-0.17766	0	0	-40.4862
-0.51184	-40.1948	-23.4577	3.63878	0	-0.44437	0	0	-29.8859
1.383664	-98.6127	-78.5807	5.084493	0	-0.82024	0	0	-50.6914
0.27076	-20.8088	-41.4267	8.410299	0	-0.79761	0	0	-5.56159

r_psp_Liga	r_psp_Liga	r_psp_Con	r_psp_Con	r_psp_Con	r_psp_Con	r_psp_Con	r_psp_Con	r_psp_Con
	29.02112	-45830	-34553	4869.544	-726.878	-3106.75	-195.796	-132.016
	18.63116	-45750.9	-34475	4874.369	-725.633	-3103.42	-192.367	-132.016
	9.954917	-45788.2	-34556.5	4868.023	-728.472	-3100.44	-190.702	-132.016
	27.65505	-45859.2	-34576.4	4868.103	-726.865	-3106.77	-195.766	-132.016
	18.61977	-45773.7	-34485.6	4871.747	-724.361	-3107.51	-191.939	-132.016
	28.88586	-33008.5	-24821	3438	-467.818	-2211.46	-147.025	-76.6085
	18.75567	-32928.9	-24743.2	3442.76	-465.906	-2211.08	-149.945	-76.6085
	9.954588	-32931.2	-24700	3431.197	-467.412	-2202.07	-140.239	-76.6085
	28.32726	-33016.8	-24830	3433.906	-467.262	-2211.33	-146.947	-76.6085
	18.61815	-32939.2	-24753.5	3434.887	-465.548	-2208.2	-146.768	-76.6085
	28.66934	-41455.3	-33598.8	5534.38	-613.245	-3338.66	-202.556	-78.1593
	17.21071	-41378	-33526.7	5541.523	-611.878	-3339.26	-201.976	-78.1593
	9.954478	-41407.6	-33527.5	5534.661	-612.027	-3338.46	-200.726	-78.1593
	28.31006	-41478.2	-33625.4	5533.539	-612.349	-3341.67	-201.89	-78.1593
	18.61858	-41393.5	-33536.3	5536.919	-611.505	-3339.55	-203.001	-78.1593
	27.7138	-12655.6	-9517.26	1116.982	-175.097	-692.358	-21.1818	-15.0219
	18.63126	-12641	-9418.86	1119.544	-173.148	-692.893	-20.481	-15.0219
	9.953174	-12630.5	-9493.17	1112.968	-172.634	-687.742	-20.4333	-15.0219
	27.65542	-12669.9	-9536.6	1113.715	-173.391	-690.644	-20.603	-15.0219
	18.61801	-12587.1	-9449.69	1117.331	-172.157	-692.366	-21.5137	-15.0219
	27.50024	-40457.9	-30109.1	4500.038	-597.516	-2549.16	-86.5731	-30.8559
	18.63018	-40382.6	-30031.4	4502.327	-597.401	-2545.68	-81.1937	-30.8559
	9.954067	-40389.6	-29973.5	4494.643	-597.313	-2543.67	-78.9286	-30.8559
	27.16317	-40478.9	-30129.7	4500.111	-597.547	-2549.49	-86.5597	-30.8559
	18.61865	-40395.1	-30048.1	4500.553	-597.04	-2544.98	-84.9417	-30.8559
	28.36313	-10623.7	-8705.83	1227.903	-160.958	-656.856	-31.9882	-5.21211
	18.75446	-10555.2	-8634.43	1231.865	-159.481	-659.645	-34.6896	-5.21211
	9.953642	-10571.4	-8644.36	1228.367	-160.479	-654.665	-30.3652	-5.21211
	26.25863	-10636.3	-8722.82	1228.277	-160.717	-657.744	-31.1566	-5.21211
	18.56674	-10570	-8644.76	1230.939	-159.314	-661.332	-37.0346	-5.21211
	28.29915	-36133.7	-27270.1	3762.862	-532.987	-2177.21	-74.3516	-71.0338
	18.75513	-36072.6	-27205	3771.809	-533.601	-2177.21	-74.1098	-71.0338

9.954773	-36086.6	-27174.3	3765.728	-532.707	-2175	-76.0206	-71.0338
28.31951	-36161.6	-27289	3760.756	-532.922	-2177.78	-77.4271	-71.0338
18.56717	-36088.3	-27215.6	3766.799	-531.814	-2181.22	-73.9923	-71.0338
27.4366	-42411.2	-32545.8	4362.182	-651.201	-3121.47	-137.258	-105.836
18.89428	-42344.3	-32473.1	4365.48	-649.59	-3121.96	-131.731	-105.836
9.954495	-42347.9	-32423.7	4356.778	-649.322	-3118.45	-129.958	-105.836
26.39669	-42389.1	-32563.8	4358.231	-649.21	-3120.1	-135.122	-105.836
18.56714	-42363.9	-32483.8	4364.103	-648.806	-3122.05	-134.11	-105.836
28.63629	-11651.8	-8159.08	981.5449	-179.877	-667.164	-15.867	-20.6568
18.75439	-11591.7	-8096.66	989.1065	-180.854	-672.339	-15.984	-20.6568
9.954288	-11600	-8020.6	980.2489	-180.999	-670.033	-15.8666	-20.6568
28.09578	-11646.5	-8137.61	987.2049	-181.041	-666.93	-15.9275	-20.6568
18.56669	-11600.1	-8106.03	984.2451	-180.867	-671.354	-15.9777	-20.6568
27.46654	-21548.1	-15887.6	2271.379	-338.674	-1375.74	-67.9406	-31.9887
18.63037	-21463.4	-15807.2	2270.7	-336.639	-1371.73	-66.3551	-31.9887
9.953127	-21489	-15859	2264.94	-338.13	-1365.34	-63.5397	-31.9887
26.40118	-21544.2	-15998	2267.853	-337.946	-1371.77	-67.8603	-31.9887
18.61816	-21487.3	-15827.8	2267.419	-336.886	-1370.52	-65.6594	-31.9887
27.62177	-40858.6	-30436.8	4280.917	-653.316	-2436.15	-98.6967	-51.506
18.63125	-40806.8	-30374	4280.138	-653.552	-2431.4	-93.8215	-51.506
9.955103	-40826.4	-30373	4274.402	-653.988	-2427.97	-92.3579	-51.506
26.24405	-40871.3	-30452.9	4279.535	-652.558	-2436.1	-99.0452	-51.506
18.56583	-40816	-30391.4	4279.653	-652.438	-2431.77	-97.4658	-51.506
28.33662	-4090.6	-3047.38	593.7031	-47.4382	-230.697	-12.4225	-24.4398
19.47219	-4023.92	-2886.5	594.2985	-45.6852	-224.107	-11.516	-24.4398
9.954281	-4039.28	-2879.1	589.7499	-45.2877	-230.119	-12.2761	-24.4398
26.39506	-4091.74	-2954.17	589.8631	-45.6219	-228.112	-12.4655	-24.4398
18.56685	-4025.86	-2974.81	593.4496	-45.653	-228.254	-11.5442	-24.4398

r_psp_Con	r_psp_Con	r_psp_Con	r_psp_Liga	r_psp_Liga	r_psp_Liga	r_psp_Liga	r_psp_Liga	r_psp_Liga
-5570.26		-6414.92	-72.832	-88.6836	4.859055	0	-1.57907	0
-5587.55		-6409.27	-3.4652	-21.2911	8.781064	0	-0.87919	0
-5528.78		-6419.33	-40.1272	-23.3575	3.637854	0	-0.44432	0
-5574.51		-6414.93	-93.2695	-108.753	4.193919	0	-1.58484	0
-5583.3		-6420.72	-21.0591	-41.3902	8.37407	0	-0.79794	0
-4212.06		-4510.51	-71.005	-88.0554	5.829505	0	-1.5778	0
-4219.15		-4505.72	-6.74481	-26.4567	8.882972	0	-0.87859	0
-4259.96		-4516.08	-40.1686	-23.458	3.638036	0	-0.44438	0
-4205.62		-4512.98	-97.7838	-115.177	2.10862	0	-1.57529	0
-4208.07		-4515.46	-21.0867	-41.3898	8.375472	0	-0.79793	0
-3983.59		-5174.69	-73.773	-89.4568	3.419707	0	-1.57007	0
-3983.39		-5178.1	-2.18903	-24.0206	13.15326	0	-0.85297	0
-3997.73		-5187.61	-40.1296	-23.4582	3.638364	0	-0.44435	0
-3978.97		-5173.35	-97.8255	-115.173	2.121094	0	-1.57541	0
-3985.56		-5176.35	-21.0853	-41.3897	8.374927	0	-0.79794	0
-2017.68		-1333.94	-69.1218	-81.5581	5.581091	0	-1.58064	0
-2103.64		-1336.52	-3.57465	-21.2899	8.780565	0	-0.87919	0
-2017.11		-1337.34	-40.0659	-23.3589	3.639567	0	-0.44434	0
-2007.57		-1339.79	-94.0183	-112.056	4.277609	0	-1.58467	0
-2013.98		-1339.67	-21.1414	-41.3895	8.375282	0	-0.79793	0
-6628.77		-4956.01	-66.2525	-77.8257	6.550317	0	-1.57976	0
-6636.99		-4961.37	-3.57569	-21.29	8.78227	0	-0.87918	0
-6693.78		-4966.2	-40.2342	-23.3583	3.638481	0	-0.44433	0
-6627.41		-4957.51	-93.3596	-107.8	2.934128	0	-1.57722	0
-6632.78		-4956.97	-21.1048	-41.3897	8.374908	0	-0.79794	0
-1194.04		-1096.69	-71.6164	-87.277	4.630893	0	-1.56965	0
-1197.03		-1096.53	-6.80507	-26.4547	8.88221	0	-0.87859	0
-1204.21		-1100.5	-40.128	-23.4585	3.639798	0	-0.44438	0
-1195.8		-1091.15	-88.8996	-99.963	4.929884	0	-1.58719	0
-1198.69		-1094.62	-20.7893	-41.4269	8.410461	0	-0.79761	0
-5769.56		-4001.33	-70.7623	-83.8226	3.550148	0	-1.57051	0
-5778.4		-4005.07	-6.78081	-26.4559	8.882673	0	-0.87859	0

-5812.11	-4011.11	-40.0247	-23.4584	3.636875	0	-0.44437	0
-5772.04	-4002.13	-97.7591	-115.177	2.116178	0	-1.57533	0
-5777.33	-4004.04	-20.8463	-41.4265	8.409915	0	-0.79761	0
-4211.1	-6000.78	-66.4574	-79.0542	6.680449	0	-1.58004	0
-4233.19	-5994.38	-2.88852	-25.6282	11.73404	0	-0.80451	0
-4272.52	-6004.86	-39.9421	-23.3585	3.637273	0	-0.44432	0
-4177.18	-5996.09	-98.8983	-78.5727	5.080002	0	-0.82022	0
-4235.06	-5998.34	-20.7913	-41.4269	8.410051	0	-0.79762	0
-2207.87	-1382.86	-74.8498	-91.0131	3.5786	0	-1.57019	0
-2204.45	-1389.84	-6.84418	-26.4549	8.882944	0	-0.87859	0
-2281.17	-1390.96	-40.1608	-23.3576	3.638047	0	-0.44432	0
-2227.38	-1384.15	-105.574	-91.3286	4.313658	0	-0.8196	0
-2202.99	-1386.48	-20.7988	-41.4266	8.410396	0	-0.79761	0
-3446.32	-2671.26	-65.2488	-74.5121	5.434823	0	-1.57987	0
-3444.86	-2675.34	-3.62155	-21.2899	8.781651	0	-0.87917	0
-3421.46	-2674.47	-40.1897	-23.4582	3.639655	0	-0.44439	0
-3332.79	-2671.7	-98.6199	-78.5751	5.077234	0	-0.82024	0
-3453.37	-2668.47	-21.0272	-41.3903	8.375581	0	-0.79793	0
-5802.49	-5660.49	-67.8468	-78.7019	5.592672	0	-1.58164	0
-5819.44	-5663.26	-3.58568	-21.2898	8.780498	0	-0.87919	0
-5826.91	-5675	-40.0709	-23.3579	3.636484	0	-0.44431	0
-5798.04	-5660.69	-87.9816	-98.4472	4.932465	0	-1.5873	0
-5807.79	-5663.31	-20.7709	-41.4269	8.411352	0	-0.79761	0
-896.054	-425.868	-71.5997	-87.1419	4.660799	0	-1.57058	0
-991.981	-433.991	-36.5224	-23.9587	8.627959	0	-0.17766	0
-994.877	-442.932	-40.1948	-23.4577	3.63878	0	-0.44437	0
-988.625	-428.165	-98.6127	-78.5807	5.084493	0	-0.82024	0
-899.362	-435.244	-20.8088	-41.4267	8.410299	0	-0.79761	0

r_psp_Liga	r_psp_Liga	r_psp_Liga	r_psp_Liga	r_psp_Rec	r_psp_Rec	r_psp_Rec	r_psp_Rec	r_psp_Rec
0	-16.4495		29.02112	-45719.6	-34440.3	4863.056	-724.358	-3092.59
0	-8.70711		18.63116	-45719.6	-34440.3	4863.056	-724.358	-3092.59
0	-29.9182		9.954917	-45719.6	-34440.3	4863.056	-724.358	-3092.59
0	-14.7804		27.65505	-45719.6	-34440.3	4863.056	-724.358	-3092.59
0	-5.86479		18.61977	-45719.6	-34440.3	4863.056	-724.358	-3092.59
0	-16.0871		28.88586	-32902.5	-24698.9	3423.771	-465.004	-2186.09
0	-7.04816		18.75567	-32902.5	-24698.9	3423.771	-465.004	-2186.09
0	-29.8588		9.954588	-32902.5	-24698.9	3423.771	-465.004	-2186.09
0	-11.4671		28.32726	-32902.5	-24698.9	3423.771	-465.004	-2186.09
0	-5.8926		18.61815	-32902.5	-24698.9	3423.771	-465.004	-2186.09
0	-14.8351		28.66934	-41345.3	-33489.2	5528.59	-610.871	-3327.43
0	-7.67943		17.21071	-41345.3	-33489.2	5528.59	-610.871	-3327.43
0	-29.8198		9.954478	-41345.3	-33489.2	5528.59	-610.871	-3327.43
0	-11.5084		28.31006	-41345.3	-33489.2	5528.59	-610.871	-3327.43
0	-5.89114		18.61858	-41345.3	-33489.2	5528.59	-610.871	-3327.43
0	-19.2779		27.7138	-12616	-9375.81	1105.056	-172.105	-682.531
0	-8.81742		18.63126	-12616	-9375.81	1105.056	-172.105	-682.531
0	-29.8554		9.953174	-12616	-9375.81	1105.056	-172.105	-682.531
0	-12.3104		27.65542	-12616	-9375.81	1105.056	-172.105	-682.531
0	-5.94733		18.61801	-12616	-9375.81	1105.056	-172.105	-682.531
0	-20.8975		27.50024	-40335.7	-29998.5	4490.26	-596.692	-2529.73
0	-8.81898		18.63018	-40335.7	-29998.5	4490.26	-596.692	-2529.73
0	-30.0241		9.954067	-40335.7	-29998.5	4490.26	-596.692	-2529.73
0	-14.0799		27.16317	-40335.7	-29998.5	4490.26	-596.692	-2529.73
0	-5.91072		18.61865	-40335.7	-29998.5	4490.26	-596.692	-2529.73
0	-15.7637		28.36313	-10512.9	-8599.78	1221.575	-158.912	-644.732
0	-7.10846		18.75446	-10512.9	-8599.78	1221.575	-158.912	-644.732
0	-29.8186		9.953642	-10512.9	-8599.78	1221.575	-158.912	-644.732
0	-18.538		26.25863	-10512.9	-8599.78	1221.575	-158.912	-644.732
0	-5.54202		18.56674	-10512.9	-8599.78	1221.575	-158.912	-644.732
0	-17.2185		28.29915	-36029.6	-27171.4	3758.044	-531.798	-2163.16
0	-7.08418		18.75513	-36029.6	-27171.4	3758.044	-531.798	-2163.16

0	-29.7136	9.954773	-36029.6	-27171.4	3758.044	-531.798	-2163.16
0	-11.4425	28.31951	-36029.6	-27171.4	3758.044	-531.798	-2163.16
0	-5.59927	18.56717	-36029.6	-27171.4	3758.044	-531.798	-2163.16
0	-19.9402	27.4366	-42301.6	-32431.1	4351.696	-648.232	-3103.21
0	-7.08414	18.89428	-42301.6	-32431.1	4351.696	-648.232	-3103.21
0	-29.731	9.954495	-42301.6	-32431.1	4351.696	-648.232	-3103.21
0	-50.9821	26.39669	-42301.6	-32431.1	4351.696	-648.232	-3103.21
0	-5.544	18.56714	-42301.6	-32431.1	4351.696	-648.232	-3103.21
0	-14.4814	28.63629	-11535.9	-8049.69	975.2657	-178.296	-654.401
0	-7.14804	18.75439	-11535.9	-8049.69	975.2657	-178.296	-654.401
0	-29.9512	9.954288	-11535.9	-8049.69	975.2657	-178.296	-654.401
0	-45.8348	28.09578	-11535.9	-8049.69	975.2657	-178.296	-654.401
0	-5.55161	18.56669	-11535.9	-8049.69	975.2657	-178.296	-654.401
0	-22.0583	27.46654	-21431.7	-15774.6	2259.42	-336.075	-1356.75
0	-8.86454	18.63037	-21431.7	-15774.6	2259.42	-336.075	-1356.75
0	-29.8798	9.953127	-21431.7	-15774.6	2259.42	-336.075	-1356.75
0	-50.703	26.40118	-21431.7	-15774.6	2259.42	-336.075	-1356.75
0	-5.83273	18.61816	-21431.7	-15774.6	2259.42	-336.075	-1356.75
0	-20.7777	27.62177	-40756.7	-30332.6	4270.25	-651.503	-2415.48
0	-8.82842	18.63125	-40756.7	-30332.6	4270.25	-651.503	-2415.48
0	-29.8602	9.955103	-40756.7	-30332.6	4270.25	-651.503	-2415.48
0	-19.1236	26.24405	-40756.7	-30332.6	4270.25	-651.503	-2415.48
0	-5.5236	18.56583	-40756.7	-30332.6	4270.25	-651.503	-2415.48
0	-15.8847	28.33662	-3984.33	-2926.32	584.9682	-44.4664	-221.021
0	-40.4862	19.47219	-3984.33	-2926.32	584.9682	-44.4664	-221.021
0	-29.8859	9.954281	-3984.33	-2926.32	584.9682	-44.4664	-221.021
0	-50.6914	26.39506	-3984.33	-2926.32	584.9682	-44.4664	-221.021
0	-5.56159	18.56685	-3984.33	-2926.32	584.9682	-44.4664	-221.021

r_psp_Rec	r_psp_Rec	r_psp_Rec	r_psp_Rec	r_psp_Rec	r_psp_MM	r_psp_MM	r_psp_MM	r_psp_MM
-188.768	-132.016	-5598.16		-6406.49	-41.1945	-23.7372	6.20E-09	-2.51947
-188.768	-132.016	-5598.16		-6406.49	-31.7519	-13.0364	-4.00E-10	-1.2746
-188.768	-132.016	-5598.16		-6406.49	-29.5992	-92.5092	3.30E-10	-4.1141
-188.768	-132.016	-5598.16		-6406.49	-48.7136	-27.4354	7.25E-09	-2.50654
-188.768	-132.016	-5598.16		-6406.49	-33.3762	-3.82101	-2.42E-09	-0.00334
-136.579	-76.6085	-4261.38		-4501.74	-43.4044	-32.7892	-0.00192	-2.81397
-136.579	-76.6085	-4261.38		-4501.74	-32.6438	-17.4348	-0.00192	-0.9016
-136.579	-76.6085	-4261.38		-4501.74	7.675901	22.37927	-0.00192	-2.40728
-136.579	-76.6085	-4261.38		-4501.74	-23.5592	-13.7807	-0.00192	-2.2576
-136.579	-76.6085	-4261.38		-4501.74	-17.8314	-12.9919	-0.00192	-0.54326
-200.276	-78.1593	-3987.28		-5180.63	-38.0312	-20.2887	8.79E-10	-2.3742
-200.276	-78.1593	-3987.28		-5180.63	-30.9528	-11.7976	2.70E-09	-1.00698
-200.276	-78.1593	-3987.28		-5180.63	-24.2865	-14.4217	2.59E-09	-1.15623
-200.276	-78.1593	-3987.28		-5180.63	-37.1705	-20.867	-4.33E-09	-1.47805
-200.276	-78.1593	-3987.28		-5180.63	-27.486	-5.69918	-3.81E-09	-0.63424
-20.4308	-15.0219	-2121.1		-1334.05	21.68087	-58.3486	-3.20E-09	-2.99235
-20.4308	-15.0219	-2121.1		-1334.05	-24.2735	-18.9301	2.00E-10	-1.04307
-20.4308	-15.0219	-2121.1		-1334.05	21.83197	-93.708	-2.97E-09	-0.5289
-20.4308	-15.0219	-2121.1		-1334.05	31.71634	-59.9019	-5.38E-09	-1.28588
-20.4308	-15.0219	-2121.1		-1334.05	47.19396	-32.1644	-3.60E-09	-0.05247
-78.1069	-30.8559	-6640.27		-4951.73	-54.6227	-19.7907	-3.50E-10	-0.82442
-78.1069	-30.8559	-6640.27		-4951.73	-45.9548	-10.5527	-5.50E-09	-0.70903
-78.1069	-30.8559	-6640.27		-4951.73	-14.4128	48.40862	8.91E-11	-0.62105
-78.1069	-30.8559	-6640.27		-4951.73	-55.9591	-21.688	-2.39E-09	-0.85534
-78.1069	-30.8559	-6640.27		-4951.73	-40.1382	-7.99413	-3.10E-09	-0.34857
-29.6458	-5.21211	-1206.22		-1090	-41.9164	-19.8016	-9.70E-10	-2.04615
-29.6458	-5.21211	-1206.22		-1090	-37.6667	-8.99593	-4.10E-09	-0.56932
-29.6458	-5.21211	-1206.22		-1090	-20.5203	-20.8959	-1.71E-09	-1.56746
-29.6458	-5.21211	-1206.22		-1090	-37.1167	-22.6912	-2.69E-09	-1.80474
-29.6458	-5.21211	-1206.22		-1090	-37.3393	-3.38504	3.24E-09	-0.40195
-72.6589	-71.0338	-5781.69		-3995.94	-35.0979	-14.136	-3.28E-09	-1.18965
-72.6589	-71.0338	-5781.69		-3995.94	-40.0881	-7.40368	-5.80E-09	-1.80341

-72.6589	-71.0338	-5781.69	-3995.94	-20.6477	20.72797	-3.10E-09	-0.9091
-72.6589	-71.0338	-5781.69	-3995.94	-35.7178	-1.92423	-2.13E-09	-1.12486
-72.6589	-71.0338	-5781.69	-3995.94	-38.2468	-2.77815	-2.93E-09	-0.01697
-127.841	-105.836	-4245.08	-5992.06	-49.0335	-36.9743	2.50E-09	-2.96885
-127.841	-105.836	-4245.08	-5992.06	-41.5124	-17.5616	4.00E-09	-1.35739
-127.841	-105.836	-4245.08	-5992.06	-7.0069	30.84715	-8.40E-10	-1.08961
-127.841	-105.836	-4245.08	-5992.06	8.131912	-53.4899	1.49E-09	-0.97734
-127.841	-105.836	-4245.08	-5992.06	-43.687	-10.8834	-1.00E-10	-0.57415
-15.8666	-20.6568	-2216.92	-1375.37	-43.0006	-18.7416	7.80E-10	-1.5804
-15.8666	-20.6568	-2216.92	-1375.37	-53.0091	-20.5747	5.00E-10	-2.55748
-15.8666	-20.6568	-2216.92	-1375.37	-26.4188	52.29735	2.10E-10	-2.70303
-15.8666	-20.6568	-2216.92	-1375.37	-19.6619	-6.18366	8.00E-10	-2.74501
-15.8666	-20.6568	-2216.92	-1375.37	-43.9522	-14.8859	2.50E-10	-2.571
-63.2925	-31.9887	-3462.71	-2665.65	-58.7253	-37.7657	-2.70E-09	-2.599
-63.2925	-31.9887	-3462.71	-2665.65	-29.9406	-10.7731	-6.60E-09	-0.56372
-63.2925	-31.9887	-3462.71	-2665.65	-18.4445	-61.2453	-2.82E-09	-2.05508
-63.2925	-31.9887	-3462.71	-2665.65	-18.5239	-144.234	-6.62E-09	-1.87068
-63.2925	-31.9887	-3462.71	-2665.65	-35.0285	-11.8092	-1.46E-09	-0.81115
-91.5743	-51.506	-5830.47	-5653.79	-40.6254	-25.0482	-2.00E-10	-1.81288
-91.5743	-51.506	-5830.47	-5653.79	-47.6521	-19.7996	-1.59E-09	-2.04875
-91.5743	-51.506	-5830.47	-5653.79	-30.3738	-17.0518	2.56E-09	-2.4847
-91.5743	-51.506	-5830.47	-5653.79	-32.5788	-21.6972	-5.82E-09	-1.05466
-91.5743	-51.506	-5830.47	-5653.79	-39.1589	-17.3813	4.80E-10	-0.93545
-10.8518	-24.4398	-905.335	-436.87	-40.5624	-33.9716	4.20E-10	-2.97174
-10.8518	-24.4398	-905.335	-436.87	-3.63567	63.92897	6.50E-10	-1.21878
-10.8518	-24.4398	-905.335	-436.87	-15.2913	70.78214	5.00E-10	-0.82127
-10.8518	-24.4398	-905.335	-436.87	-10.2052	50.65095	-8.00E-11	-1.15546
-10.8518	-24.4398	-905.335	-436.87	-21.0886	-7.09408	3.50E-10	-1.18657

r_psp_MM	r_psp_MM	r_psp_MM	r_psp_MM	r_psp_MM	r_psp_MM	r_psp_Rec	r_psp_Rec	r_psp_Rec
-12.6169	-7.02736	0	44.07689		-39.3705	0	0	0
-9.94318	-3.59866	0	19.12794		-23.027	0	0	0
-7.40352	-1.93389	0	99.2123		-22.8508	0	0	0
-12.612	-6.99743	0	38.28988		-37.4521	0	0	0
-14.1169	-3.17024	0	20.65428		-32.919	0	0	0
-23.6104	-10.4461	0	64.54835		-38.2911	0	0	0
-24.1261	-13.3664	0	48.95409		-25.7671	0	0	0
-15.5293	-3.65979	0	31.35928		-24.4643	0	0	0
-23.5186	-10.3682	0	65.72573		-39.3579	0	0	0
-21.2943	-10.1892	0	59.16132		-31.9722	0	0	0
-9.64603	-2.28028	0	19.04242		-22.4845	0	0	0
-11.0212	-1.70067	0	11.18509		-16.6115	0	0	0
-10.5816	-0.45011	0	19.26184		-16.9388	0	0	0
-12.6392	-1.61408	0	19.91483		-20.4871	0	0	0
-11.323	-2.72482	0	7.611136		-14.7159	0	0	0
-8.28194	-0.75099	0	122.1295		-30.0748	0	0	0
-9.49135	-0.05021	0	25.0111		-19.7699	0	0	0
-4.76245	-0.0025	0	133.787		-12.9531	0	0	0
-6.53611	-0.17221	0	131.4263		-31.8139	0	0	0
-9.01397	-1.08285	0	112.91		-23.4024	0	0	0
-17.8506	-8.46615	0	26.96457		-34.6555	0	0	0
-15.0636	-3.08682	0	11.69208		-28.2348	0	0	0
-13.4963	-0.82168	0	-23.6474		-24.235	0	0	0
-18.1952	-8.45282	0	26.76416		-33.532	0	0	0
-14.4439	-6.83483	0	13.32845		-23.8452	0	0	0
-10.5989	-2.34245	0	28.34553		-35.4728	0	0	0
-14.0317	-5.0438	0	16.50435		-25.5303	0	0	0
-9.47385	-0.71943	0	31.83214		-19.6958	0	0	0
-11.4298	-1.51082	0	29.02197		-28.7021	0	0	0
-15.7932	-7.38885	0	13.03952		-23.4097	0	0	0
-12.4911	-1.69268	0	29.1122		-34.7006	0	0	0
-13.1816	-1.45092	0	10.03507		-26.2836	0	0	0

-11.3935	-3.36164	0	-0.63119	-25.0802	0	0	0
-13.0593	-4.76816	0	20.95382	-35.7952	0	0	0
-17.2564	-1.33342	0	9.926185	-26.7881	0	0	0
-16.651	-9.41733	0	54.70749	-37.7295	0	0	0
-17.8966	-3.89003	0	19.31842	-20.1252	0	0	0
-14.7892	-2.11719	0	2.424421	-22.2825	0	0	0
-16.0821	-7.28101	0	118.1843	-32.2221	0	0	0
-18.0159	-6.26889	0	15.70822	-23.6528	0	0	0
-11.1665	-0.00042	0	23.3727	-34.8843	0	0	0
-17.0817	-0.11744	0	19.19404	-31.8718	0	0	0
-15.1871	-6.85E-08	0	-34.2877	-26.5384	0	0	0
-11.7191	-0.06091	0	38.88426	-37.8376	0	0	0
-16.1552	-0.11111	0	19.48895	-29.7179	0	0	0
-17.4204	-4.64806	0	38.68111	-34.9732	0	0	0
-14.0883	-3.06263	0	26.58759	-28.0405	0	0	0
-8.14635	-0.24721	0	71.30184	-18.0523	0	0	0
-14.2062	-4.56776	0	180.3587	-34.0044	0	0	0
-12.9688	-2.36691	0	15.2047	-22.2772	0	0	0
-19.0822	-7.1224	0	48.68806	-36.2478	0	0	0
-15.0391	-2.24718	0	19.80825	-28.3257	0	0	0
-12.0573	-0.78364	0	33.5371	-31.5335	0	0	0
-19.0285	-7.47096	0	51.80865	-35.1361	0	0	0
-15.4871	-5.8915	0	28.59412	-28.0577	0	0	0
-8.12473	-1.57066	0	25.97809	-19.9018	0	0	0
-2.9079	-0.66417	0	-46.2321	-16.5417	0	0	0
-8.64985	-1.42434	0	-59.6739	-15.504	0	0	0
-6.28134	-1.61373	0	-32.7319	-19.0737	0	0	0
-6.43445	-0.69238	0	11.53043	-17.2116	0	0	0

r_psp_Rec	r_psp_Rec	r_psp_Rec	r_psp_Rec	r_psp_Rec	r_psp_Rec	r_psp_Rec_Strain_vdW
0	0	0	0	0		0
0	0	0	0	0		0
0	0	0	0	0		0
0	0	0	0	0		0
0	0	0	0	0		0
0	0	0	0	0		0
0	0	0	0	0		0
0	0	0	0	0		0
0	0	0	0	0		0
0	0	0	0	0		0
0	0	0	0	0		0
0	0	0	0	0		0
0	0	0	0	0		0
0	0	0	0	0		0
0	0	0	0	0		0
0	0	0	0	0		0
0	0	0	0	0		0
0	0	0	0	0		0
0	0	0	0	0		0
0	0	0	0	0		0
0	0	0	0	0		0
0	0	0	0	0		0
0	0	0	0	0		0
0	0	0	0	0		0
0	0	0	0	0		0
0	0	0	0	0		0
0	0	0	0	0		0
0	0	0	0	0		0
0	0	0	0	0		0
0	0	0	0	0		0
0	0	0	0	0		0
0	0	0	0	0		0

0	0	0	0	0	0
0	0	0	0	0	0
0	0	0	0	0	0
0	0	0	0	0	0
0	0	0	0	0	0
0	0	0	0	0	0
0	0	0	0	0	0
0	0	0	0	0	0
0	0	0	0	0	0
0	0	0	0	0	0
0	0	0	0	0	0
0	0	0	0	0	0
0	0	0	0	0	0
0	0	0	0	0	0
0	0	0	0	0	0
0	0	0	0	0	0
0	0	0	0	0	0
0	0	0	0	0	0
0	0	0	0	0	0
0	0	0	0	0	0
0	0	0	0	0	0
0	0	0	0	0	0
0	0	0	0	0	0
0	0	0	0	0	0
0	0	0	0	0	0
0	0	0	0	0	0
0	0	0	0	0	0
0	0	0	0	0	0
