# Supplementary material for: Protective Mechanisms of Nootropic Herb Shankhpushpi (Convolvulus pluricaulis) against Dementia: Network Pharmacology and Computational Approach
Source: Evid Based Complement Alternat Med. 2022 Oct 3;2022:1015310. doi: 10.1155/2022/1015310 (PMC9550454; doi:10.1155/2022/1015310)
Supplement: Supplementary Materials — Supplementary Figure S1. Protein-protein interaction network. Node size is proportional to its degree of connectivity. Supplementary Table S1. Target genes for molecular docking. Supplementary Table S2. QikProp ADMET of CP compounds. Supplementary Table S3. Targets for CP compounds. Supplementary Table S4 data for CP compound-target-disease network. Supplementary Table S5. MM-GBSA free energy calculation result. [file 1015310.f1.zip › Supplemetary Figure S1.docx]

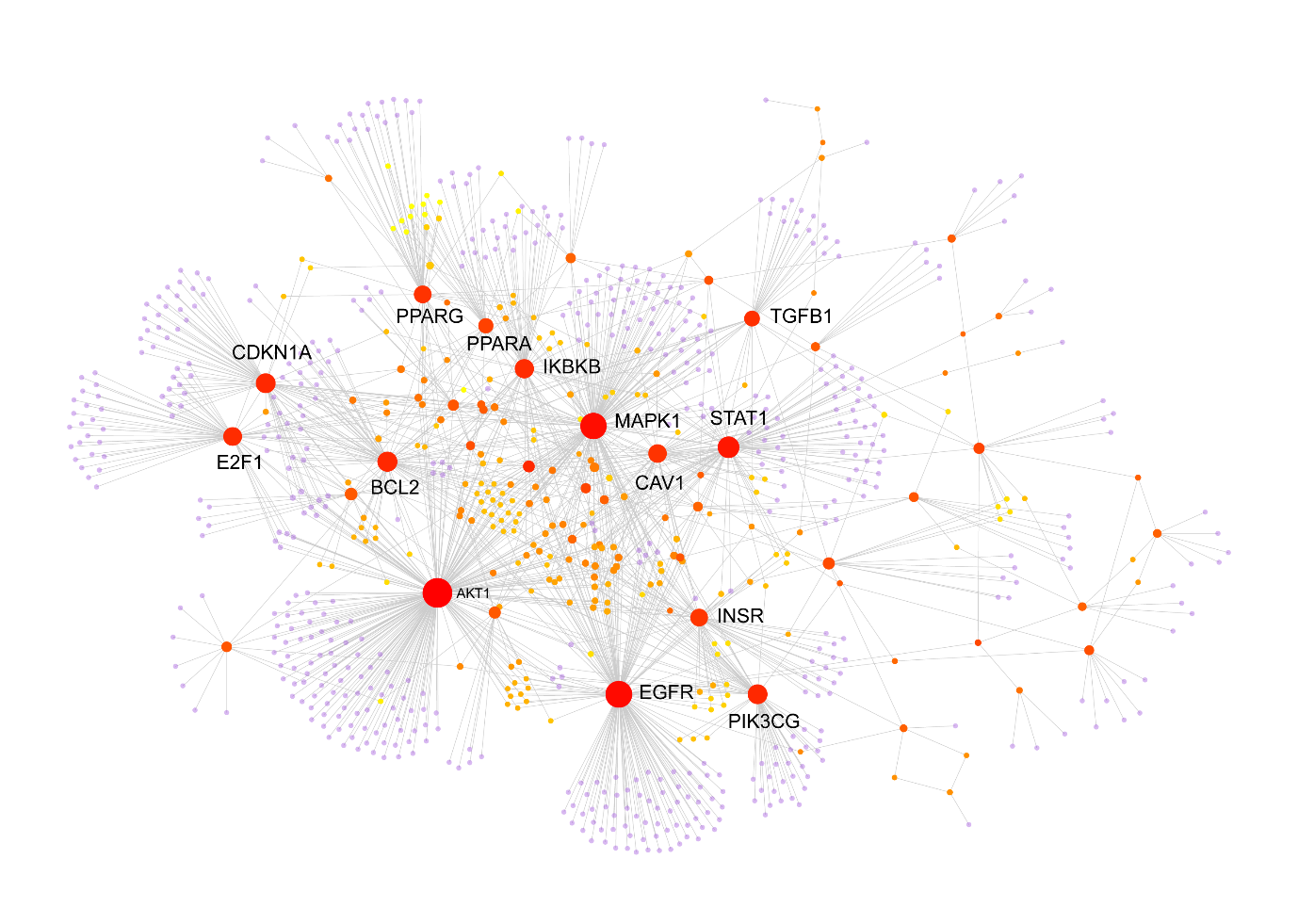


**FIGURE S1.** Protein-protein interaction network. Node size is proportional to its degree of connectivity.
